# Associations between perinatal risk and physical health in pre-adolescence in the Adolescent Brain Cognitive Development (ABCD) Study®: the unexpected relationship with sleep disruption

**DOI:** 10.1038/s41390-024-03288-z

**Published:** 2024-06-08

**Authors:** Shana Adise, Clare E. Palmer, Chandni Sheth, Andrew T. Marshall, Fiona C. Baker, Sandra A. Brown, Linda Chang, Duncan B. Clark, Rada K. Dagher, Vanessa Diaz, Frank Haist, Megan M. Herting, Rebekah S. Huber, Kimberly LeBlanc, Karen C. Lee, Huajan Liang, Janosch Linkersdörfer, Krista M. Lisdahl, Jiyoung Ma, Gretchen Neigh, Megan W. Patterson, Perry Renshaw, Kyung E. Rhee, Calen Smith, Susan F. Tapert, Wesley K. Thompson, Kristina A. Uban, Deborah Yurgelun-Todd, Elizabeth R. Sowell

**Affiliations:** 1 Division of Endocrinology, Diabetes, Metabolism, Department of Pediatrics, Children’s Hospital Los Angeles, University of Southern California, Los Angeles, CA USA; 2 Center for Human Development, University of California San Diego, La Jolla, CA USA; 3 Department of Psychiatry, University of Utah School of Medicine, Salt Lake City, UT USA; 4 Division of Neurology, Children’s Hospital Los Angeles, Department of Pediatrics, University of Southern California, Los Angeles, CA USA; 5 Center for Health Sciences, SRI International, Menlo Park, CA USA; 6 Department of Psychology, University of California San Diego, La Jolla, CA USA; 7 Department of Psychiatry, University of California San Diego, La Jolla, CA USA; 8 Diagnostic Radiology and Nuclear Medicine, University of Maryland, Baltimore, MD USA; 9 Departments of Psychiatry and Pharmaceutical Sciences, University of Pittsburgh, Pittsburgh, PA USA; 10 Division of Clinical and Health Services Research, National Institute on Minority Health and Health Disparities, Bethesda, MD USA; 11 Division of Extramural Research, National Institute on Drug Abuse, Bethesda, MD USA; 12 Department of Population and Public Health Sciences, University of Southern California, Los Angeles, CA USA; 13 Eunice Kennedy Shriver National Institute of Child Health and Human Development, Bethesda, MD USA; 14 Department of Psychology, University of Wisconsin, Milwaukee, WI USA; 15 Department of Anatomy and Neurobiology, Virginia Commonwealth University, Richmond, VA USA; 16 University of Colorado Denver - Anschutz Medical Campus, Denver, CO USA; 17 Department of Pediatrics, University of California San Diego, La Jolla, CA USA; 18 Laureate Institute for Brain Research, Tulsa, OK USA; 19 Health Society & Behavior, Program of Public Health, University of California, Irvine, CA USA

## Abstract

**Background:**

To investigate relationships among different physical health problems in a large, sociodemographically diverse sample of 9-to-10-year-old children and determine the extent to which perinatal health factors are associated with childhood physical health problems.

**Methods:**

A cross-sectional study was conducted utilizing the Adolescent Brain Cognitive Development℠ (ABCD) Study (n = 7613, ages 9-to-10-years-old) to determine the associations among multiple physical health factors (e.g., prenatal complications, current physical health problems). Logistic regression models controlling for age, sex, pubertal development, household income, caregiver education, race, and ethnicity evaluated relationships between perinatal factors and childhood physical health problems.

**Results:**

There were significant associations between perinatal and current physical health measures. Specifically, those who had experienced perinatal complications were more likely to have medical problems by 9-to-10 years old. Importantly, sleep disturbance co-occurred with several physical health problems across domains and developmental periods.

**Conclusion:**

Several perinatal health factors were associated with childhood health outcomes, highlighting the importance of understanding and potentially improving physical health in youth. Understanding the clustering of physical health problems in youth is essential to better identify which physical health problems may share underlying mechanisms.

**Impact:**

- Using a multivariable approach, we investigated the associations between various perinatal and current health problems amongst youth.
- Our study highlights current health problems, such as sleep problems at 9-to-10 years old, that are associated with a cluster of factors occurring across development (e.g., low birth weight, prenatal substance exposure, pregnancy complications, current weight status, lifetime head injury).
- Perinatal health problems are at large, non-modifiable (in this retrospective context), however, by identifying which are associated with current health problems, we can identify potential targets for intervention and prevention efforts.

## Introduction

The developmental origins of health and disease framework has long been leveraged to guide examination of how perinatal factors have lasting impact on later developmental and adult health outcomes.^1^ For example, lower birth weight is associated with later independent infant milestones, such as a greater likelihood of late onset of sitting and walking,^2^ and higher risk of cardiovascular problems later in life.^3^ The causes of low infant birth weight are multifactorial, but, in particular, prenatal substances of abuse exposure (PSE) is thought to be one contributing factor.^4–6^ PSE has also been identified as an independent risk factor for childhood obesity,^7^ other cognitive (e.g., impulsivity and attention problems, poorer cognitive functioning)^8^ and health outcomes health (e.g., growth restriction sleep disturbances^9–12^). Moreover, each of these aforementioned health outcomes impart their own risk for other adverse health outcomes. For example, inadequate sleep in childhood is associated with increased risk for childhood obesity and neurological and psychiatric disorders.^13–16^ Meanwhile, childhood obesity is associated with sleep apnea^17^ and sleep disturbances,^18^ and prolonged obesity over time increases the incidence of type 2 diabetes.^19^ Thus, clusters of perinatal problems may have lasting effects on a complex web of comorbid health outcomes in childhood. As such, understanding whether different health factors or exposures during the perinatal period are related to health outcomes in childhood, and how these childhood health outcomes are interrelated may be insightful for predicting, and potentially preventing, subsequent physical health (PH) challenges. Collectively, this information may reveal common targets for intervention that can more effectively minimize subsequent health problems.

Previous studies have attempted to examine the interrelatedness and comorbidities between PH measures and behavioral outcomes. However, these foundational studies have traditionally only considered one or two PH measures. Here, we utilize a large and diverse data set with multiple assessments of PH problems across the perinatal (retrospective recall) and childhood period to determine if there is indeed interrelatedness and comorbidity of PH problems across the lifespan that are important for predicting health outcomes at 9/10-years-old. To do this, we utilized data from the Adolescent Brain Cognitive Development℠ Study (ABCD Study®, hereafter, “ABCD”): a large, longitudinal study designed to understand factors that promote healthy development and/or place youth at risk for health problems.^20^ In doing so, we may better understand the shared variability among PH measures which will enable interpretation of the interactions between the measures and resultant clinical outcomes. Specifically, ABCD’s baseline PH measures include retrospective caregiver-report perinatal factors (e.g., PSE, birth, and pregnancy complications), developmental milestones, health behaviors such as physical activity, and current PH outcomes like sleep disturbances, medical problems, and body mass index (BMI). Our previous report details the extent of PH problems found in youth enrolled in the ABCD study.^21^ The current manuscript aims to: 1) examine the co-occurrence of current and retrospective parent-report PH issues in the ABCD Study sample in late childhood; 2) to identify the interrelatedness of retrospective and current PH problems (i.e., baseline assessment); 3) examine the associations among perinatal (retrospective recall) and current PH outcomes; and 4) to understand the extent to which perinatal factors were associated with PH problems 9-to-10-years later. Understanding these relationships at 9/10 years old (i.e., the baseline assessment in the ABCD Study) will allow for future insight into how these developmental trajectories change throughout the course of adolescence.

## Methods

### Participants

ABCD is a 10-year longitudinal study of 11,878 children across 21 US sites; the inclusion/exclusion criteria have been described elsewhere.^21^ Recruitment aimed to mirror demographic variation in the US population.^22^ All children provided verbal assent while the attending caregiver provided written consent. The ABCD Study had a centralized Institutional Review Board approved by the University of California San Diego. The current study included a sub-sample of youth at baseline (aged 9-to-10-years old, *n* = 7613; release 2.0.1 10.15154/1522647) with complete measures across PH variables (i.e., to ensure each model included the same sample for comparison of effects). Importantly, only one youth per family was randomly included, so that there were no issues of dependence, and more computationally efficient models could be utilized.^23^ Participant demographics are displayed in Table 1.

**Table 1:** Demographics of the sample.

	Analyzed Sample	Excluded Sample	*p* value
*n*	7613	2340	
Age at interview [mean (SD)]	118.72 (7.39)	118.98 (7.35)	0.135
Sex at birth = Male [*n* (%)]	3973 (52.2)	1250 (53.6)	0.248
Household income [*n* (%)]			<0.001
[<50 K]	2182 (28.7)	595 (40.6)	
[>=50 K and <100 K]	2165 (28.4)	393 (26.8)	
[>=100 K]	3266 (42.9)	479 (32.7)	
Highest parental education [*n* (%)]			<0.001
<HS Diploma	274 (3.6)	238 (10.2)	
HS Diploma/GED	615 (8.1)	359 (15.4)	
Some College	1939 (25.5)	656 (28.2)	
Bachelor	2018 (26.5)	449 (19.3)	
Post Graduate Degree	2767 (36.3)	622 (26.8)	
Caregiver self-declared race [*n* (%)]			<0.001
White	5136 (67.5)	1104 (50.4)	
Black	1038 (13.6)	536 (24.5)	
Asian	165 (2.2)	85 (3.9)	
Other/Mixed	1274 (16.7)	465 (21.2)	
Latinx or Hispanic ethnicity = Yes [*n* (%)]	1488 (19.5)	633 (28.6)	<0.001
Perceived pubertal developmental stage [*n* (%)]			0.001
Pre	3940 (51.8)	891 (46.4)	
Early	1792 (23.5)	500 (26.1)	
Mid	1763 (23.2)	496 (25.8)	
Late/Post	118 (1.5)	32 (1.7)	

Sub-sample from the ABCD Study with complete data across all measures analyzed and with one participant from each family to produce an independent sample. The number and proportion of participants across demographics of age, assigned sex at birth, household income, highest parental education, youth’s caregiver-reported race and ethnicity, and perceived pubertal developmental stage (as indicated by the Pubertal Development Scale). Sociodemographics were compared between the analyzed sample (*n* = 7613) and the remainder of the independent sample excluded for not having complete cases across all measures analyzed (*n* = 2340). The analyzed sample had a significantly different distribution for all demographics apart from age and sex at birth.

### Physical health measures

The Supplementary Materials Methods section contains details of these measures, while the distribution of PH outcomes is detailed elsewhere.^21^ Below, each measure is described in brevity.

#### Perinatal measures

The Developmental History Questionnaire,^24–26^ completed by the caregiver at the baseline assessment (ages 9/10-years-old), collected retrospective information on birthweight, gestational age, developmental milestones, medical problems during pregnancy and birth, including questions pertaining to PSE. Endorsement of the following was counted as a medical problem during pregnancy: a) severe nausea and vomiting beyond 6 months or accompanied by weight loss; b) heavy bleeding requiring bed rest or special treatment; c) (pre)eclampsia/toxemia; d) severe gallbladder attack; e) persistent proteinuria; f) rubella during first 3 months of pregnancy; g) severe anemia; h) urinary tract infections; i) pregnancy-related diabetes; j) pregnancy-related high blood pressure; k) problems with placenta; and l) accident or injury requiring medical care. Endorsement of the following was counted as a medical problem during birth: a) blue-at-birth; b) slow heartbeat; c) not breathing at first; d) convulsions; e) jaundice, f) needing treatment; g) required oxygen; h) required blood transfusion; and i) Rh incompatibility.

#### Current PH measures

Caregivers and youth completed other questionnaires to provide information on current PH outcomes and behaviors at the baseline visit (ages 9/10-years-old) including past medical history and retroactive reports of lifetime medical problems (e.g., asthma, hospitalizations, surgeries), history of traumatic brain injury (TBI), sleep disturbances (Sleep Disturbance Scale for Children [SDSC]), sleep duration, physical activity, and sports participation. Notably, lifetime medical problems included any medical problem from infancy through current age, excluding TBI and birth and pregnancy complications. The lifetime medical problems and TBI were treated as a relatively “current” PH factor because it includes retrospective caregiver report of medical information from birth up to 9-to-10 years. Body mass index (BMI; kg/m^2^) collected from height and weight were utilized to classify participants as underweight (<5th %ile), healthy weight (≥5th %ile to <85th %ile), overweight (≥85th %ile to <95th %ile), or obese (≥95th %ile), using the CDC 2000 Growth Chart SAS (SAS Institute, Inc.; Cary, NC).^27^ Pubertal development was measured using the caregiver report Pubertal Development Scale (PDS).^20^

### Clinical guidelines/recommendation classification

To determine whether participant’s PH data fell within or beyond clinical guidelines/recommendation thresholds, the scores for each PH measurement that had relevant “clinical guidelines” was transformed into a binary variable (0 [fell within guidelines/recommendations] or 1 [fell outside guidelines/recommendations]). For example, youth who were prenatally exposed to substances of abuse (PSE) received a 1 in this domain, whereas those who were not, were coded as a 0 (fell within guidelines/recommendations. Table 2 summarizes binarization cutoffs and the number of participants classified as 0 (met clinical guidelines/recommendations) and 1 (did not meet clinical guidelines/recommendations) for each PH measurement.

**Table 2:** Physical health (PH) measures.

Measure	Reporter	Developmental Timing	Not Endorsed (Coded = 0)	Endorsed (Coded = 1)	*N* = 0	*N* = 1	% Endorsed
Prenatal Alcohol Exposure	Caregiver	Perinatal	No exposure pre or post pregnancy recognition	Exposure either only pre-pregnancy recognition or both pre- and post-pregnancy recognition	5503	2110	27.7
Prenatal Tobacco Exposure	Caregiver	Perinatal	No exposure pre or post pregnancy recognition	Exposure either only pre-pregnancy recognition or both pre- and post-pregnancy recognition	6638	975	12.8
Prenatal Marijuana Exposure	Caregiver	Perinatal	No exposure pre or post pregnancy recognition	Exposure either only pre-pregnancy recognition or both pre- and post-pregnancy recognition	7171	442	5.8
Prenatal Other Substances Exposure	Caregiver	Perinatal	No exposure pre or post pregnancy recognition	Exposure either only pre-pregnancy recognition or both pre- and post-pregnancy recognition	7503	110	1.4
Medical Problems (Pregnancy)	Caregiver	Perinatal	No medical problems during pregnancy	≥2 medical problem during pregnancy	6493	1120	14.7
Medical Problems (Birth)	Caregiver	Perinatal	No medical problems during birth	≥2 medical problem during birth	7131	482	6.3
Premature Birth	Caregiver	Perinatal	Gestational age ≥37 weeks	Gestational age <37 weeks	6794	819	10.8
Low Birthweight	Caregiver	Perinatal	Birth weight >2500 g	Birth weight ≤2500 g	6707	906	11.9
High Birthweight	Caregiver	Perinatal	Birth weight ≤4000 g	Birth weight >4000 g	6913	700	9.2
Rolling Over Age (Beyond Guidelines)	Caregiver	Perinatal	Age ≤6 months	Age >6 months	7521	92	1.2
Sitting Age (Beyond Guidelines)	Caregiver	Perinatal	Age ≤9 months	Age >9 months	7321	292	3.8
First Words Age (Beyond Guidelines)	Caregiver	Perinatal	Age ≤12 months	Age >12 months	6902	711	9.3
First Walking Age (Beyond Guidelines)	Caregiver	Perinatal	Age ≤18 months	Age >18 months	7378	235	3.1
Sleep Duration (Short)	Caregiver	Current	8–9 and 9–11 average hours sleep per night	<7 h or 7–8 h average sleep per night	6502	1111	14.6
High score for sleep disturbance	Caregiver	Current	Total sleep disturbance scale for children (SDSC) score <39	Total sleep disturbance scale for children (SDSC) score ≥39	5203	2410	31.7
Physical Activity—Vigorous (Below Guidelines)	Youth	Current	≥60 mins vigorous physical activity per day	<60 min vigorous physical activity per day	1282	6331	83.2
Physical Activity—Strengthening (Below Guidelines)	Youth	Current	≥3 days per week of strengthening exercises	<3 days per week strengthening exercises	2350	5263	69.1
Sports Activities (No Participation)	Caregiver	Current	Some participation (>0 average hours per week)	No participation (0 average hours per week)	5936	1677	22.0
BMI (Underweight)	Objective	Current	No underweight (≥5th BMI percentile)	Underweight (<5th BMI percentile)	7331	282	3.7
BMI (Obese)	Objective	Current	No obesity (<95th BMI percentile)	Obese (≥95th BMI percentile)	6357	1256	16.5
Medical Problems (Lifetime)	Caregiver	Current	No medical problems reported throughout lifetime	≥2 medical problems reported throughout lifetime	4591	3022	39.7
Traumatic brain injury (TBI)	Caregiver	Current	Improbable TBI	Possible, mild, moderate, or severe TBI	7314	299	3.9

PH measures collected in the ABCD Study with thresholds used for binarization. “Developmental Timing” refers to the time in development for which the physical health characteristic is relevant: the first 13 variables describe the perinatal period; the latter 9 variables describe the current developmental stage or are lifetime measures. The penultimate 2 columns from the right show the number of participants who experienced (coded = 1) or did not experience (coded = 0) the PH problem. The final column shows the percentage of participants who endorsed that problem. *BMI* body mass index.

### Statistical analysis

#### Co-occurrence of PH measures

To determine the number of co-occurring PH clinical guidelines/recommendations, a summary score was calculated across all PH assessments, where 0 indicated no co-occurring PH measures, while 12 indicated co-occurrence across all PH measures.

#### Interrelatedness of PH measures

Correlations were conducted to determine the likelihood of participants not meeting clinical guidelines/recommendations for more than 1 PH measure.

#### Examination of associations amongst PH measures

Logistic regression models were conducted to understand if there was a relationship between each PH measure. Models were controlled for youth’s sex-at-birth, caregiver-reported race and ethnicity, household income, caregiver education, and study site; models where current PH measures were the dependent variable (DV) also controlled for age and PDS. Notably, PDS was removed as a covariate in the model when BMI was the DV due to the lack of underweight participants who were late- or post-pubertal. Odds ratios (OR), confidence intervals (CI), and *p*-values are reported. In total, 458 regression models were conducted and corrected using a Bonferroni adjusted alpha value of 0.05/458 = 0.00011. Associations were evaluated in MATLAB using custom code that will be available here on publication: https://github.com/ABCD-STUDY. Importantly, our analyses chose to control for race and ethnicity, due to the fact that underrepresented racial and ethnic groups are at increased risk for experiencing disparities in physical health due to greater exposure to disadvantage from structural inequalities in the US. Therefore, race and ethnicity were included as covariates to statistically account for potential differences in physical health attributable to social determinants (i.e., structural racism).^21,28^ Inferences regarding health disparities are beyond the scope of this manuscript, and as such, associations with sociodemographic covariates will not be discussed.

#### Multivariable post-hoc associations between sleep and other PH measures

Because we observed strong relationships between retrospective perinatal/current PH measures and high sleep disturbances, we conducted post-hoc analyses to probe these associations further. Multiple 5-fold cross-validated regression models were conducted in R 3.6.3 to estimate the unique variance in sleep measures explained (*R*^2^) by retrospective perinatal and current PH measures; models were controlled for age and PDS. By including all measures together in multiple regression models, we were able to measure the variability in sleep measures (parent and child report) predicted by retrospective perinatal or current PH variables, while accounting for the unique and shared variability among these variables. The following models were estimated:

Model 1)

$${DV} \sim {age} 	+{PDS}+{sex}+{income}+{caregiver\; education} \\ 	+{race}+{ethnicity}+{site}+\varepsilon$$

Model 2)

$${DV} \sim {age} 	+{PDS}+{sex}+{income}+{caregiver\; education}+{race} +{ethnicity} \\ 	+{site}+{Perinatal\; PH\; measures}+{Current\; PH\; measures}+\varepsilon$$

Model 3)

$${DV} \sim {age} 	+{PDS}+{sex}+{income}+{caregiver\; education}+{race}+{ethnicity} \\ 	+{site}+{Current\; PH\; measures}+\varepsilon$$

Model 4)

$${DV} \sim {age} 	+{PDS}+{sex}+{income}+{caregiver\; education}+{race}+{ethnicity} \\ 	+{site}+{Perinatal\; PH\; measures}+\varepsilon$$

To estimate the unique variability in each sleep disturbance measure predicted by all relevant PH measures, $\triangle {R}^{2}$ was calculated as the absolute change in ${R}^{2}$ from a null model (Model 1; covariates only) to a full model (Model 2). To estimate the unique variability in each sleep disturbance measure predicted by retrospective *perinatal PH measures* only, controlling for *current PH measure*, $\triangle {R}^{2}$ was calculated as the absolute change in ${R}^{2}$ from Model 2 to Model 3. To estimate the unique variability in each DV predicted by *current PH measures*, controlling for retrospective *perinatal PH measures*, $\triangle {R}^{2}$ was calculated as the absolute change in ${R}^{2}$ from Model 2 to Model 4.

## Results

### Adherence to clinical guidelines

Only 61 (0.8%) participants were within clinical or recommended thresholds for all PH measures, that is they had no health (or risk of) problems assessed (Supplementary Fig. 1). This was largely driven by the high number of participants who did not meet the recommended clinical guidelines for physical activity (69–83%; Table 2).

### Co-occurrence of PH problems

It was most common for participants to have at least 2 PH problems and 1 PH risky behavior (e.g., low physical activity) across all measures (*n* = 1673; 21.98%; Supplementary Fig. 1). Excluding endorsement of low physical activity, 44.7% of the sample (caregiver or youth report) endorsed ≥1 perinatal PH problem and ≥1 current PH problem (at 9-to-10-years old). Only 19.13% of the sample experienced ≥1 perinatal PH problem without any current PH problems; 23.33% of the sample reported ≥1 current PH problem without any perinatal PH complications. Co-occurrences of PH problems are shown in Supplementary Figs. 1–3.

### Interrelatedness of PH problems

**Fig. 1:**
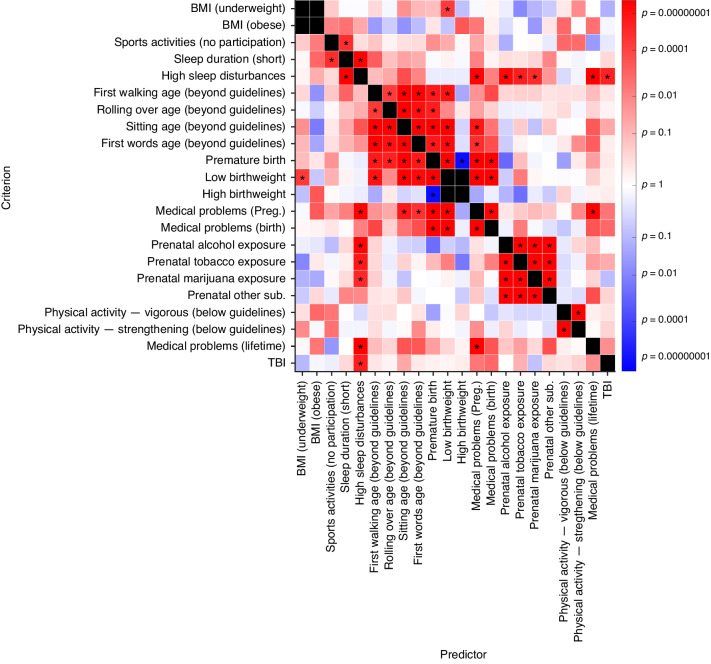
Association between likelihood of participants’ exhibiting physical health conditions. Association matrix across dichotomized physical health measures. Each column represents the predictor of interest in a binomial logistic regression analysis; each row represents the corresponding criterion. The shading of each box represents the strength of the association in terms of *p* values, with more red regions reflecting greater positive associations and more blue regions representing greater negative associations. The color bar was scaled for ease of interpretation. Associations that passed Bonferroni correction are marked by asterisks (*). All models controlled for sociodemographic factors of sex, household income, caregiver education, race, and ethnicity, with models looking at a current criterion measure also including age and pubertal status.

The likelihood of participants not being within clinical guidelines/recommendations for more than one measure is presented in Fig. 1 and Supplementary Table 1.

### The association among perinatal PH measures

Perinatal measures were significantly associated with one another. Caregiver reported premature birth was associated with increased odds of having a low birthweight (*OR* [95% CI] = 26.26 [21.61, 31.91]), delayed developmental milestones (rolling over: OR = 3.54 [2.16, 5.81]; sitting: *OR* = 3.76 [2.83, 4.99]; walking: *OR* = 2.51 [1.78, 3.55]; first spoken word: *OR* = 1.85 [1.48, 2.31]), and experiencing ≥2 medical problems during pregnancy (OR = 3.65 [3.07, 4.36]) or birth (*OR* = 5.73 [4.58, 7.16]). Among participants whose caregiver endorsed any perinatal complications (*n* = 4,856), only 835 participants (17.2%) experienced birth or pregnancy problems without associated low birthweight, prematurity, or developmental delays. Experiencing ≥2 medical problems during pregnancy was associated with delays in first sitting (OR = 2.14 [1.62, 2.82]); however, the endorsement of delayed sitting was low (*n* = 292; 3.8%).

### The association between perinatal and childhood PH measures

The only childhood PH measure to show associations with perinatal PH problems was caregiver-reported sleep disturbances; interestingly, this was the most common childhood PH problem (*n* = 2,410; 31.7%). Nearly 61.2% of caregivers who reported their youth had high sleep disturbances also endorsed either complications during pregnancy or birth or ≥2 lifetime medical problems (not including perinatal problems); 15.3% endorsed both lifetime and perinatal medical problems. An increased likelihood of high sleep disturbances at 9-to-10-years-old was significantly associated with caregiver report of medical problems during pregnancy (*OR* = 1.63 [1.42, 1.86]), PSE (alcohol: *OR* = 1.45 [1.30, 1.63]; tobacco: *OR* = 1.46 [1.27, 1.70]; marijuana: *OR* = 1.62 [1.32, 1.98]), and shorter sleep duration (*OR* = 3.14 [2.72, 3.61]).

### The association among childhood PH measures

Sleep disturbances was the only childhood PH measure that showed multiple associations with other PH measures at 9-to-10-years old. The likelihood of high sleep disturbances was increased ~80% by TBI (*OR* = 1.84 [1.45, 2.34] and 100% by experiencing ≥2 lifetime medical problems (*OR* = 2.08 [1.88, 2.31]). Of all participants whose caregivers reported high sleep disturbances, 61.2% of them also endorsed either complications during pregnancy or birth or ≥2 lifetime medical problems (not including perinatal problems); 15.3% endorsed both lifetime and perinatal medical problems.

### Post-hoc analysis: Multivariable prediction of high sleep disturbance

**Fig. 2:**
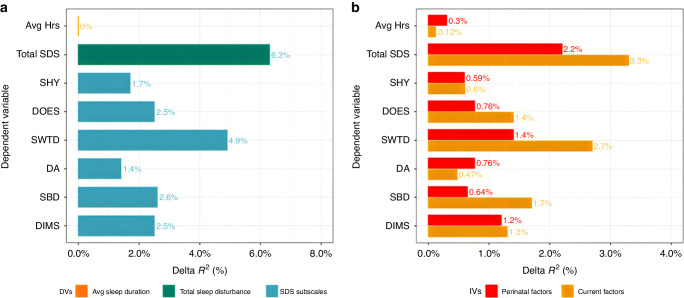
Individual variability in sleep measures predicted by physical health factors. **a** Out-of-sample cross-validated ${R}^{2}$ estimates for each DV (y-axis) uniquely predicted by all physical health factors associated with high sleep disturbances in the first analysis (developmental delay during infancy, medical problems during birth and pregnancy, prenatal substance exposure, lifetime medical problems and TBI) over and above covariates (age, PDS, assigned sex-at-birth, race, ethnicity, household income, caregiver education and data collection site). **b** Out-of-sample cross-validated ${R}^{2}$ estimates for each DV uniquely predicted by ${P}$ (red; set of perinatal factors [retrospective recall]) and $C$ (yellow; set of current factors) in a multiple regression model controlling for covariates and the other set of IVs respectively. SDSC sub-scales: SHY sleep hyperhydrosis, DOES disorders of excessive somnolence, SWTD sleep-wake transition disorders, DA disorders of arousal, SBD sleep breathing disorder, DIMS disorders of initiating and maintaining sleep, Avg Hrs average hours per night total sleep duration.

Perinatal and childhood PH measures explained 6.3% of the variability in caregiver-reported youth total sleep disturbances and 4.9% of the variability in caregiver-reported youth sleep-wake transition disorders but explained none of the variability in caregiver-reported youth average sleep duration (Fig. 2a). When analyzed separately, perinatal PH measures contributed 2.2% of the variability in caregiver reported youth total sleep disturbances, while childhood PH measures accounted for 3.3% of the variability in PH measures (Fig. 2b). The unique variability in sleep disturbance predicted by perinatal variables was relatively similar across sub-scales. However, the childhood compared to perinatal PH measures explained more unique variability in sleep-wake transition disorders. Additional associations for each PH measure and sleep disturbance subscale can be found in Supplemental Figure 4.

When accounting for the shared variability among PH measures, endorsing ≥2 medical problems, having a TBI, and endorsing ≥2 problems during pregnancy showed the largest associations with sleep disturbances followed by endorsing ≥2 problems during birth. The association between a high number of concurrent medical problems and sleep-wake transition disorders may be contributing to the higher variability in sleep-wake transition disorders predicted by childhood PH variables. Supplementary analyses included weight status as an additional IV given the trending association between obesity and high sleep disturbances (Supplementary Figs. 5, 6). Including weight status in statistical analyses only slightly increased the variability explained by total sleep disturbance scores but did increase the variability in sleep-disordered breathing explained by childhood PH factors.

## Discussion

In a large sample of 9-to-10-year-old youth from the ABCD Study, we found high co-occurrence across caregiver report of retrospective perinatal and current childhood health outcomes. However, only caregiver report of high sleep disturbances at 9-to-10-years old was associated with retrospective perinatal PH and current childhood health outcomes. Caregiver endorsement of PSE, at least two problems during birth and pregnancy, and being delayed in first sitting and speaking each increased the likelihood of having increased sleep disturbances at 9-to-10-years old by ~30–60%. Together, these factors explained ~2% of the variability in total sleep disturbances. Endorsing at least two lifetime medical problems and/or having a history of TBI were each associated with ~80% increase in the likelihood of having increased sleep disturbances and together predicted an additional ~3% of the individual variability in total sleep disturbances at 9/10-years old. The high co-occurrence in PH problems suggests that there may be similar underlying mechanisms among their development. However, similar to how low birth weight can result from many different prenatal factors, these results could reflect other exposures or underlying mechanisms not captured in the ABCD measures. These results also highlight high sleep disturbances as a potential predictive factor and/or target for intervention to improve future health outcomes. However, because variability in sleep disturbances was uniquely predicted by retrospective perinatal and current childhood PH problems, there may be multiple pathways contributing to the development of sleep disturbances in late childhood.

In line with previous research,^29–33^ we found significant associations across retrospective perinatal health factors; for example, caregiver report of youth premature delivery was significantly correlated with having low birthweight, experiencing at least two medical complications during birth and/or pregnancy, and delayed developmental milestones. This highlights the importance of controlling for shared variability amongst these measures, especially when investigating associations between specific retrospective perinatal factors and later PH outcomes. Interestingly, the variability in sleep disturbances at 9/10-years old predicted by retrospective perinatal factors was independent of the variability predicted by current childhood health factors. This generates novel hypotheses about potentially differential causal pathways that could contribute to the presence of sleep problems at 9-to-10-years old.

Few previous studies have shown associations between perinatal complications and subsequent sleep disturbances in this age group. However, caregiver report of in-utero exposure to preeclampsia was associated with obstructive sleep apnea and sleep-disordered breathing in childhood.^34^ PSE has also been shown to correlate with sleep problems from ages 3-to-12 years.^12,35,36^ It is possible that different perinatal factors disrupt sleep via different pathways and therefore contribute uniquely to sleep disturbance sub-scales. However, the current data showed that perinatal factors predicted a similar proportion of variability and a similar pattern of associations across sleep disturbance sub-scales (although we did not statistically test for uniformity in our analyses). These findings suggest that the perinatal factors studied may contribute to a global disruption of sleep-wake neural systems. In this sample, caregiver endorsement of different perinatal factors was relatively low, so it is difficult to determine which specific perinatal problems contributed the most variability to later sleep disturbances. Studies with greater detail of perinatal data will be useful to determine targets specifically for intervention.

The association between lifetime medical problems and sleep disturbance is not surprising given that sleep disorders in childhood are often comorbid with asthma, allergies, and bronchitis.^37^ Additionally, these were the most common medical problems experienced in the ABCD sample.^21^ There is also growing evidence of a relationship between high sleep disturbances and TBI.^38^ Here, across SDSC sub-scales, current PH factors explained the most unique variability in sleep-wake transition disorders. Meanwhile, endorsing ≥2 current medical problems and having a TBI showed the largest associations with sleep-wake transition disorders. Sleep-wake transition disorders are classified as parasomnias that occur during the transition from wakefulness to sleep or from one sleep stage to another. These disorders include rhythmic movement disorder, sleep talking, and nocturnal leg cramps.^39^ The neurobiological mechanisms underlying sleep-wake transition disorders are unclear, but potentially include complex interactions between subcortical neuromodulatory circuits and the cerebral cortex.^40^ It is conceivable that medical problems, especially a TBI, may impair neuronal signaling in the neurocircuits involved in sleep and wakefulness. However, caregivers were assessed generally about TBI, and did not provide specific dates or youth age at time of the event. Therefore, it is unknown as to when TBI occurred relative to the onset of sleep problems. Additionally, it is also possible that daytime sleepiness or certain sleep disorders (e.g., sleepwalking) could increase the risk of TBI.

Interestingly, PH factors predicted little variability in sleep duration at 9-to-10-years old. Perhaps this is because sleep duration is strongly associated with sociodemographic factors, such as household income.^41^ Here, in the current analyses, PH factors were not associated with unique variability in sleep duration beyond that explained by sociodemographic factors. Further, external environmental factors that influence sleep quality were not collected nor included in the present analyses. Thus, the degree to which the observed associations between sleep and PH factors occur on an individual, rather than environmental, level remains unknown and warrant further investigation.

Unexpectedly, not meeting recommended guidelines for physical activity was not associated with any of the PH measures, although there was a trending association with increased weight status. In our sample, a large proportion of participants did not meet recommended CDC guidelines for physical activity. So, it is possible that our assessment of physical activity may not be sensitive enough to capture the relationship between extreme inactivity and risk of experiencing co-occurring PH problems. Notably, because youth reported physical activity, this may not be an accurate assessment and as such, explain the lack of sensitivity. However, it could also be that the associations between low physical activity and perinatal and current PH problems may be more apparent as the youth transition into puberty. Future analyses with continuous measures of physical activity may reveal important associations not detectable here.

## Strengths and limitations

This study used a large population-based sociodemographically diverse sample of children, which strengthens the generalizability of inferences and the specificity and statistical power of the analyses. There is substantial value of simultaneous consideration of a broad array of personal/biological, behavioral, and environmental factors in a single study of youth in this age range. Our findings provide insights for future examination of sex-specific variability in emergent physical health and mental health problems typically unfolding in adolescence. However, there are a few limitations. For example, we used race and ethnicity as a distal measure of potential health disparities, due to systemic inequities, so our inferences are limited about health disparities; future studies should include a wider set of environmental and social variables that encompass social determinants of health. Relatedly, we note the limitations of BMI z-scores which were used to categorize youth into weight class groups^42^ and future studies should incorporate more accurate measures of body composition. The questionnaires are also well-established with good validity and reliability. However, questionnaire-based data collection in absence of objective measures of PH is a limitation. Moreover, use of caregiver-report measures regarding developmental and medical history is subject to recall bias, and caregiver-reported sleep disturbance among youth may not accurately reflect the child’s sleep disturbance. The availability of electronic health records with future ABCD Study data releases will be crucial for validating these measures in the current sample. In the current study we generated relatively coarse binary variables for ease of computation and to create clinically relevant dichotomous measures. Future studies in developmental samples enriched for perinatal complications with more fine-grained data will be useful for generating more specific hypotheses about the factors most important for predicting later health outcomes. Perinatal factors examined have some clear overlap in mechanisms (e.g., preeclampsia/eclampsia overlaps with high proteinuria and elevated blood pressure), thus greater details on perinatal factors in data collection would allow for better disentangling of commonly co-occurring perinatal factors). We cannot infer any causal effects due to the cross-sectional and observational nature of the study, though future analyses with the longitudinal data in ABCD may be enlightening. Finally, we cannot examine other possible key factors important for PH measures like sleep that were not measured in the ABCD Study (e.g., noise/light pollution), therefore, additional studies are needed to assess these finer-grained mechanisms.

## Conclusions

Overall, the current study found a high prevalence of health problems (that were highly interrelated) in a socio-demographically diverse sample of 9-to-10-year-old children in the US. There was a high co-occurrence of PH problems particularly with sleep disturbances. Improvements in perinatal care and education and/or treatment to improve sleep quality in children may improve later health outcomes across domains. This study highlights the importance of leveraging the dense phenotyping within the ABCD Study to understand the shared variance across multiple factors contributing to health outcomes across the developmental trajectory.

## Supplementary information

Supplementary Materials

## Data Availability

The ABCD data repository grows and changes over time. The ABCD data used in this report came from NIMH Data Archive Digital Object Identifier 10.15154/1522647. DOIs can be found at 10.15154/1522647.
