# Supplementary material for: Associations between perinatal risk and physical health in pre-adolescence in the Adolescent Brain Cognitive Development (ABCD) Study®: the unexpected relationship with sleep disruption
Source: Pediatr Res. 2024 Jun 8;96(7):1834–42. doi: 10.1038/s41390-024-03288-z (PMC11772240; doi:10.1038/s41390-024-03288-z)
Supplement: Supplementary file 1 — Supplementary Materials [file 41390_2024_3288_MOESM1_ESM.pdf]

SUPPLEMENTARY MATERIALS

- Supplementary Methods | Pg 2
- Supplemental References | Pg 10
- Supplementary Figure 1: Co-occurrence of all physical health problems | Pg 11
- Supplementary Figure 2: Co-occurrence of perinatal physical health problems | Pg 12
- Supplementary Figure 3: Co-occurrence of current physical health problems | Pg 13
- Supplementary Figure 4. Multiple regression statistics predicting sleep measures | Pg 14
- Supplementary Figure 5. Individual variability in sleep measures predicted by physical health factors including weight status | Pg 15
- Supplementary Figure 6. Multiple regression statistics predicting sleep measures including weight status | Pg 16
- Supplementary Table 1. Odds ratios, 95% confidence intervals (CI) and p-values for logistic regression models | Pg 17

Supplementary Methods

Physical Health Measures

Physical health measures were comprised of questionnaires evaluating past and current physical health. In addition, some objective measures including height, weight, waist circumference were collected at the time of the baseline visit.

Perinatal/developmental history

The Developmental History Questionnaire, originally developed by the Adolescent Component of the National Comorbidity Survey[1,2] was completed by the caregiver at baseline to obtain information on birth weight, gestational age, early developmental milestones, medical problems during birth and pregnancy, and prenatal substance exposure.

Birth weight and prematurity. Caregivers were asked whether their child was born prematurely and, if yes, how many weeks premature. Birth weight (pounds, ounces) was converted to kilograms (kg) and categorized based on the Centers for Disease Control guidelines as extremely low birth weight (ELBW, < 1,000 g), very low birth weight (VLBW, 1000 to < 1,500 g), low birth weight (LBW, 1500 to < 2,500 g), average/normal birth weight (NBW, 2500 to < 4,000 g), and high birth weight (HBW, > 4,000 g). One exclusionary criterion for study participation was a birth weight of <1200 g; however, there are a few participants who did report this low birth weight. Nevertheless, given the low number of participants in the ELBW category, we combined the ELBW and VLBW categories for analysis. Generally, an infant is defined as premature if birth occurred before 37 weeks of gestation. Consistent with the current literature, we further categorized participants into extremely preterm (EPI, if born <28 weeks), very preterm (VPI, 28 to <32 weeks), moderate preterm (MPI, 32 to <34 weeks), and late preterm

(LPI, 34 to 37 weeks).[3] One child born before 35 weeks and weighing 14 pounds was removed, likely due to measurement error.

Early developmental milestones. Caregivers were asked about the age at which their child began to roll over (delayed if after 6 months), sit without assistance (delayed if after 9 months), walk without assistance (delayed if after 18 months), and say his/her first word (delayed if after 12 months.[4] Outliers due to measurement error were excluded from analysis by setting an upper threshold of 48 months for rolling over, sitting, and walking and 60 months for first word. The questionnaire also assessed caregiver concern regarding motor and speech delays, with caregivers asked to compare their child's development to that of other children (earlier, average, later).

Medical problems during birth and pregnancy. Caregivers were asked about any complications during birth and pregnancy using dropdown lists. Medical problems during birth were blue-at-birth, slow heartbeat, did not breathe at first, convulsions, jaundice needing treatment, required oxygen, required blood transfusion, and Rh incompatibility. Medical problems during pregnancy were severe nausea and vomiting extending past the 6th month accompanied by weight loss, heavy bleeding requiring bed rest or special treatment, (pre)eclampsia/toxemia, severe gall bladder attack, persistent proteinuria, rubella during first 3 months of pregnancy, severe anemia, urinary tract infections, pregnancy related diabetes, pregnancy related high blood pressure, problems with the placenta, accident or injury, or any other conditions. A summary variable was calculated for each of these as a Total Problems score based on a sum of the number of complications endorsed (separately for birth and pregnancy). A categorical variable was created to summarize those who had no problems, one problem, or more than one problem (i.e., ≥2).

Prenatal substance exposure. Caregivers were asked about the biological mother's substance use (i.e., tobacco, alcohol, marijuana, cocaine/crack, heroin/morphine, oxycontin, and any other drugs) before the mother found out about the pregnancy (but could have been pregnant) and once the mother knew about the pregnancy. If drug use was endorsed, follow-up questions were asked about the frequency and quantity of use. In addition to the above drugs of abuse, caffeine use from conception until delivery was also measured. Here, we measured endorsement of use of each substance before and after pregnancy by combining the two variables asking about pre- and post-pregnancy recognition of substance exposure into a single variable with three categories: 1) pre-recognition no use + post-recognition no use; 2) pre-recognition use + post-recognition no use; 3) pre-recognition use + post-recognition use. There were 59 subjects who endorsed no use before pregnancy recognition but use after recognition; these subjects were excluded from analysis given the small number in this category. This graded exposure variable dependent on timing of pregnancy recognition was computed for alcohol, tobacco, cannabis (i.e., marijuana), and other substance exposure.

Current measures

Caregivers and youth completed several other questionnaires about the child's current physical health.

Sleep. The Sleep Disturbance Scale for Children (SDSC) was used to assess sleep duration and sleep disturbance symptoms at the baseline visit.[5] The 26-item SDSC assesses frequency of disorders of initiating and maintaining sleep, sleep breathing disorders, disorders of arousal, sleep-wake transition disorders, disorders of excessive somnolence, and sleep hyperhidrosis in the past 6 months. We used the overall sleep-wake disturbance score, which was the sum of all items, with higher scores reflecting a greater clinical severity of sleep disturbance.

We excluded 8 subjects who scored greater than 87, which was the maximum score attained in the original study. A cut-off score of 39 is recommended as a threshold for identifying children with disturbed sleep. The individual item from the SDSC, "How many hours of sleep does your child get on most nights?"' was used as the measure for typical total sleep duration. Possible responses were: 1) 9-11 hours, 2) 8-9 hours, 3) 7-8 hours, 4) 5-7 hours, and 5) less than 5 hours. As very few participants endorsed fewer than 5 hours of sleep per night, the smallest two categories were combined to form a category of "less than 7 hours". Responses of "7-8 hours" and the collapsed category of "less than 7 hours" constituted short sleep durations.

Physical activity and sports activity: Three items from the Youth Risk Behavior Survey (YRBS) served as a measure of physical activity. YRBS was modified from the Youth Risk Behavior Survey.[6,7] Youth were asked the number of days in the past week that they exercised for at least 60 minutes per day and the number of days in the past week that they engaged in exercises to strengthen or tone their muscles. The questionnaire also asks about how many days per week the youth had physical education (PE) class in school. The Sports and Activities Involvement Questionnaire, modeled after the assessment developed for the Vermont Health and Behavioral Questionnaire and the Dutch Health Behavioral Questionnaire [8], measured lifetime and past year involvement in 23 different sports, activities like music and dance, and other hobbies. A summary score of time spent participating in sports (excluding all items associated with activities or hobbies that do not require physical activity) was calculated for each participant. For each sport, caregivers reported: 1) time spent (mins) per session (*tspent*); 2) number of days per week when participating (*perwk*); 3) number of months per year (*nmonth*); 4) number of years participated (at baseline visit); and, 5) whether the child participated in the last 12 months. For each sport endorsed in the past 12 months, the mean participation hours per

week was calculated using the following formula: past year mean hours per week per sport = (*tspent* × *perwk* × *nmonth*)/52)/60. This value was summed across all sports endorsed to create a total average time spent participating in sports over the past year for each participant.

BMI/Weight Status: Anthropometric measurements of height and weight were taken as the average of up to 3 separate measures using professional grade equipment (e.g., physician weight beam scale with height rod). Body mass index (BMI) (kg/m^2) was converted to age- and sex-specific percentiles using the CDC 2000 Growth Chart SAS (SAS Institute, Inc, Cary, NC).[8] The CDC age- and sex-adjusted percentiles were used to classify participants as underweight (i.e., $<5^{th}$ %ile) healthy weight ($\geq 5^{th}$ %ile to $< 85^{th}$ %ile), overweight ($\geq 85^{th}$ %ile to $<95^{th}$ %ile), obese ($\geq 95^{th}$ %ile). Subjects with potential measurement error who had biologically implausible BMIs [e.g., extremely small BMI ($n = 23$) and extremely large BMI ($n = 5$)] were excluded. These extreme values were identified using cut offs of <-4 and >8 of the modified BMI z-scores, which express an individual's BMI relative to the median BMI at that age and sex. These scores were calculated using SAS code from the CDC: https://www.cdc.gov/nccdphp/dnpao/growthcharts/resources/sas.htm#reference. Statistical analyses were conducted using the above noted weight status classifications.

Medical Problems (lifetime)*:* A caregiver-report medical history questionnaire about the youth was derived from the Missouri Assessment of Genetics Interview for Children Health Services Utilization Questionnaire.[9] At baseline, the questionnaire covered both past year and lifetime conditions including the following: asthma, allergies, bronchitis, leukemia, cerebral palsy, diabetes, epilepsy, hearing loss, kidney disease, lead poisoning, muscular dystrophy, multiple sclerosis, vision problems, heart problems, sickle cell anemia, headache, operation, and other illnesses. A *Total Problems* summary score was created by summing endorsed conditions

for each participant. A categorical variable was created based on whether participants endorsed none, one, or more than one problem (i.e., ≥ 2). Participants endorsing more than 6 medical problems were excluded due to questions regarding the validity of the data ($n = 47$).

Traumatic Brain Injury or Head injury (lifetime): Caregivers reported on the youth's lifetime history of head injury using the Modified Ohio State University TBI Screen-Short Version. [10,11] This questionnaire asks whether their child had been to the emergency room due to an injury to the head or neck and whether the child had injured their neck in a fall or from being hit by something or from being in a fight or from a gunshot wound. A positive response to an occurrence question was followed up with questions to determine loss of consciousness (LOC), memory loss, and other details about the event (e.g., age at time of injury). A summary variable with the worst injury overall is generated as follows: Improbable TBI (responses to all head injuries are "no"); TBI without LOC or memory loss (response to at least one question about head injury is "yes" but all responses to LOC and memory loss are "no"); possible mild TBI (TBI without LOC but with memory loss); mild TBI (TBI with LOC less than 30 minutes); moderate TBI (TBI with LOC between 30 minutes and 24 hours), or severe TBI (TBI with LOC greater than 24 hours).

Visualizing Co-occurrence of Physical Health Problems

To illustrate the amount of overlap between different perinatal and current physical health problems in the sample, we used the visualization technique "UpSet".[12] We first collapsed the 22 binary variables used in the analyses described above into 6 variables indicating perinatal (prematurity, low birth weight, high birth weight, developmental delay, prenatal substance exposure, medical problems during birth or pregnancy) as well as 6 variables indicating current/lifetime health issues (low weight status, high weight status, low physical activity, sleep

problems, lifetime medical problems, head injury). We then visualized the amount of overlap between (1) these 12 reported health issues (Supplementary Figure 1), (2) perinatal health problems (Supplementary Figure 2), and (3) current health issues (Supplementary Figure 3). In these figures, we restricted the number of displayed overlaps to those that were reported by at least 30 participants, therefore the total sum of all the bars plotted is less than the total sample size. Plotting was done using R version 4.0.4 (2021-02-15) (R Core Team, 2021) and the ComplexUpset package (version 1.3.0).

Binarization of variables: Such binarization was used across domains as several variables had skewed distributions, were highly zero-inflated, or had very low endorsement (e.g., having had leukemia). This is a limitation of our approach.

Analyses

Multivariable post-hoc associations between sleep and other PH measures. Because we observed strong relationships between perinatal/current PH measures and high sleep disturbances, we conducted postdoc analyses to probe these associations further. As such, we quantified the variability across SDSC sub-scales (e.g., sleep hyperhidrosis, disorders of excessive somnolence, sleep-wake transition disorders, disorders of arousal, sleep breathing disorders, and disorders of initiating and maintaining sleep) with other PH factors. We also included average nightly sleep duration as a DV. Multiple 5-fold cross-validated regression models were conducted to estimate the variance explained (R^2) for each model out-of-sample (i.e., in a test dataset). To estimate the unique variability in sleep measures predicted by perinatal and current variables, respectively, we compared multiple regression models with a set of perinatal variables, *P = (medical problems [birth], medical problems [pregnancy], PSE,*

developmental milestones), and a set of current PH variable, *C = (medical problems [lifetime], TBI)*, controlling for covariates including age and PDS. By including all measures together in multiple regression models, we were able to measure the variability in sleep measures (parent and child report) predicted by perinatal or current PH variables, while accounting for the unique and shared variability among these variables. Medical problems at birth, during pregnancy, and across the lifetime were coded with three levels to allow for a gradation of medical problems: 0, 1, or >1.

Supplementary References

1. Kessler, R.C., et al., *Design and field procedures in the US National Comorbidity Survey Replication Adolescent Supplement (NCS-A).* International Journal of Methods in Psychiatric Research, 2009. **18**(2): p. 69-83.
2. Kessler, R.C., et al., *National Comorbidity Survey Replication Adolescent Supplement (NCS-A): II. Overview and design.* Journal of the American Academy of Child & Adolescent Psychiatry, 2009. **48**(4): p. 380-385.
3. Chung, E.H., J. Chou, and K.A. Brown, *Neurodevelopmental outcomes of preterm infants: a recent literature review.* Translational Pediatrics, 2020. **9**: p. S3-S8.
4. Karcher, N.R., et al., *Assessment of the prodromal questionnaire-brief child version for measurement of self-reported psychoticlike experiences in childhood.* JAMA Psychiatry, 2018. **75**(8): p. 853-861.
5. Bruni, O., et al., *The Sleep Disturbance Scale for Children (SDSC). Construction and validation of an instrument to evaluate sleep disturbances in childhood and adolescence.* Journal of Sleep Research, 1996. **5**(4): p. 251-261.
6. Kann, L., et al., *Youth Risk Behavior Surveillance - United States, 2015.* Morbidity and Mortality Weekly Report: Surveillance Summaries, 2016. **65**(6): p. 1-174.
7. Barch, D.M., et al., *Demographic, physical and mental health assessments in the adolescent brain and cognitive development study: Rationale and description.* Developmental Cognitive Neuroscience, 2018. **32**: p. 55-66.
8. Huppertz, C., et al., *Individual differences in exercise behavior: Stability and change in genetic and environmental determinants from age 7 to 18.* Behavior Genetics, 2016. **46**: p. 665-679.
9. Barlow, S.E. and W.H. Dietz, *Obesity evaluation and treatment: Expert committee recommendations.* Pediatrics, 1998. **102**(3): p. e29.
10. Freedman, D.S., et al., *Validity of the WHO cutoffs for biologically implausible values of weight, height, and BMI in children and adolescents in NHANES from 1999 through 2012.* The American Journal of Clinical Nutrition, 2015. **102**(5): p. 1000-1006.
11. Todd, R.D., et al., *Reliability and stability of a semistructured DSM-IV interview designed for family studies.* Journal of the American Academy of Child & Adolescent Psychiatry, 2003. **42**(12): p. 1460-1468.
12. Corrigan, J.D. and J. Bogner, *Initial reliability and validity of the Ohio State University TBI identification method.* Journal of Head Trauma Rehabilitation, 2007. **22**(6): p. 318-329.
13. Bogner, J.A., et al., *Test-retest reliability of traumatic brain injury outcome measures: A traumatic brain injury model systems study.* Journal of Head Trauma Rehabilitation, 2017. **32**(5): p. E1-E16.
14. Lex, A., et al., *UpSet: Visualization of intersecting sets.* IEEE Transactions on Visualization and Computer Graphics, 2014. **20**(12): p. 1983-1992.

A

no. of overlapping health problems	n	%
0	61	0.80
1	384	5.04
2	1215	15.96
3	1673	21.98
4	1560	20.49
5	1172	15.39
6	759	9.97
7	421	5.53
8	215	2.82
9	82	1.08
10	45	0.59
11	16	0.21
12	6	0.08
13	1	0.01
14	3	0.04

B

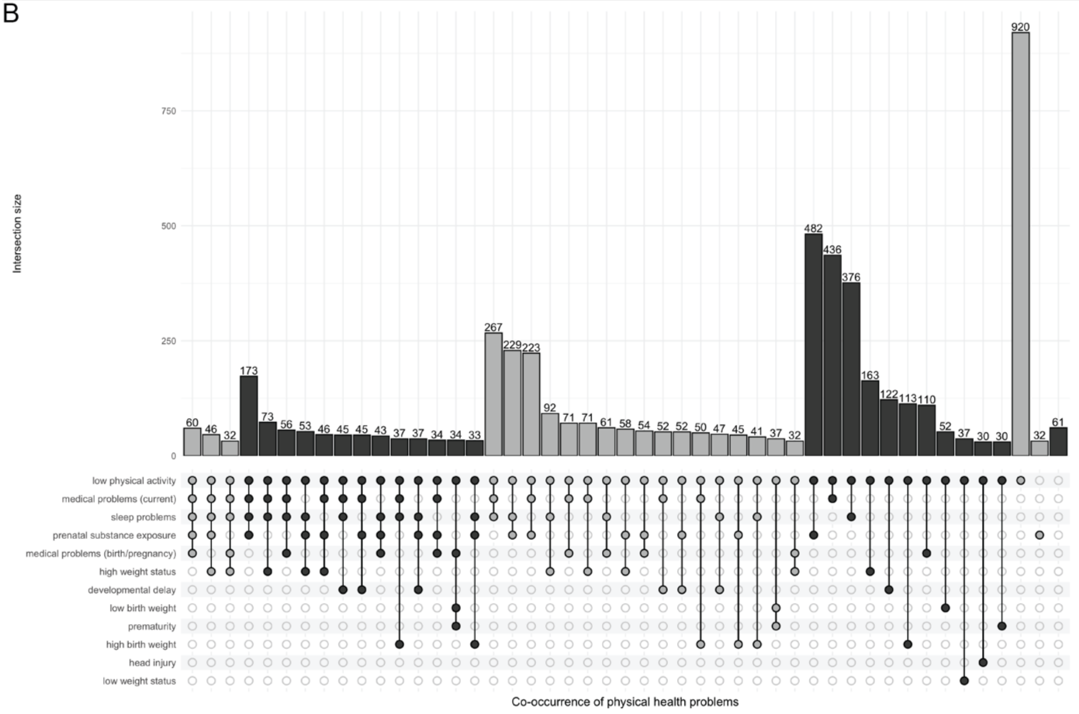

Supplementary Figure 1. Co-occurrence of all physical health problems. A) Table showing the number and proportion of participants endorsing a different number of co-ocurring physical health problems. It was most common in this sample for participants to endorse 3 co-occuring problems across developmental periods (21.98%). B) "UpSet" graph visualizing the co-occurrence of different perinatal and current physical health problems. The dots in the matrix below the bar graph indicate a given combination of different problems. The bars above the dots indicate the number of youth exhibiting the respective combination. Only combinations of health problems that were reported by at least 30 participants are shown, therefore the sum of all the bars is lower than our overall sample size. For simplicity physical health factors have been collapsed to create a lower number of measures for overlap comparison. Only 61 participants showed no endorsement of any physical health problem (far right bar). This was in part driven by the high endorsement of low physical activity across the sample and thus this appears in nearly all combinations of physical health problems. Only 920 participants experienced low physical activity without any other physical health problem (third bar from the right). The most common combination of physical health problems was low physical activity and potential prenatal substance exposure (n=482) followed by low physical activity and lifetime medical problems (n=436), followed by low physical activity and sleep problems (n=376).

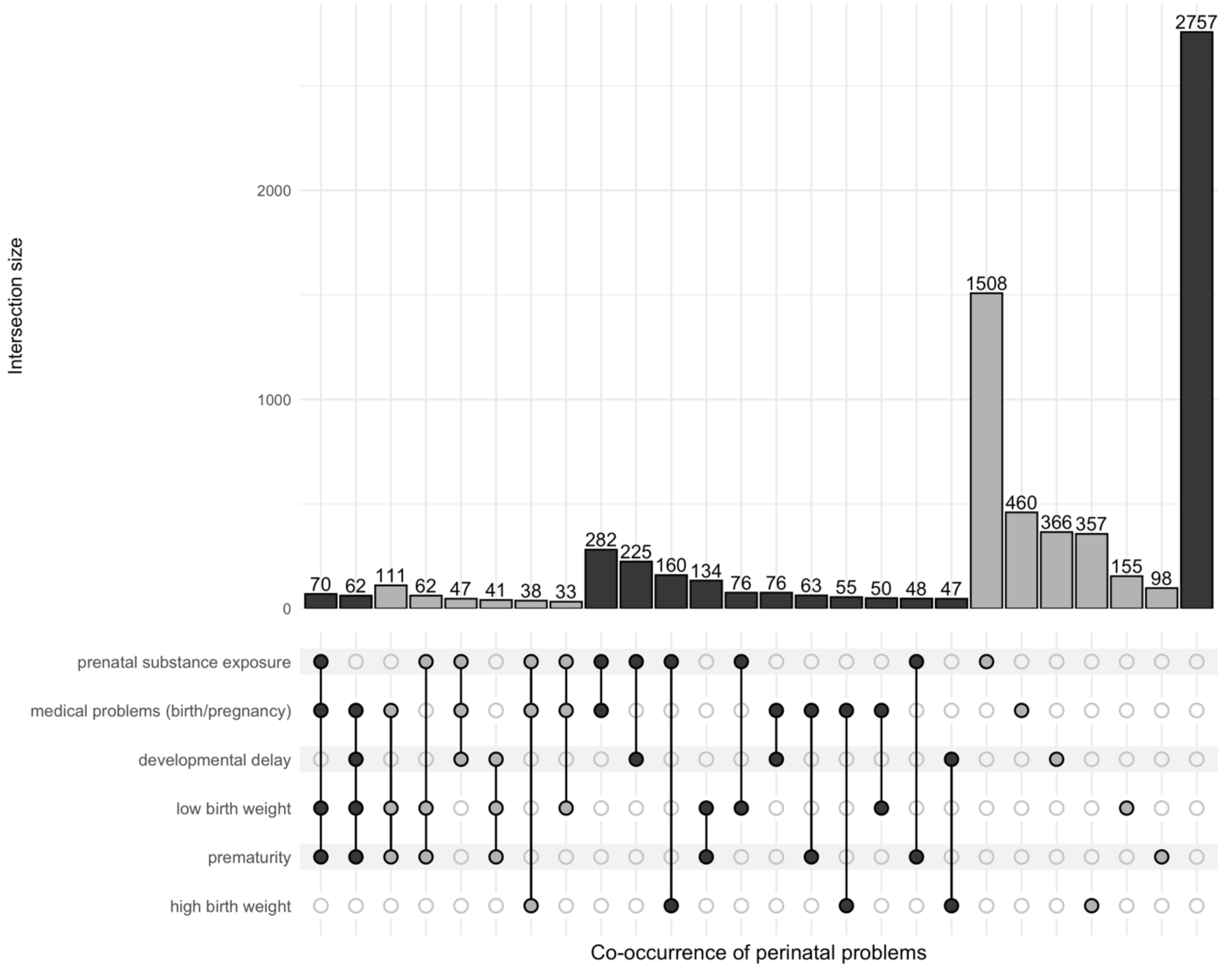

Supplementary Figure 2. Co-occurrence of perinatal physical health problems. "UpSet" graph visualizing the co-occurrence of different perinatal physical health problems. The dots in the matrix below the bar graph indicate a given combination of different problems. The bars above the dots indicate the number of youth exhibiting the respective combination. Only combinations of health problems that were reported by at least 30 participants are shown, therefore the sum of all the bars is lower than the overall sample size. For simplicity physical health factors have been collapsed to create a lower number of measures for overlap comparison. The most highly endorsed single perinatal problem was potential prenatal substance exposure (n=1508) driven by alcohol use. Importantly, this variable includes exposure either only pre-pregnancy recognition or both pre- and post-pregnancy recognition, therefore reflects potential exposure. The most common combination of co=occurring perinatal problems was prenatal substance exposure and medical probelems during birth and/or pregnancy (n=282) followed by prenatal substance exposure and developmental delay (n=225). The total number of participants who experienced any perinatal problem was 4856 (63.79%).

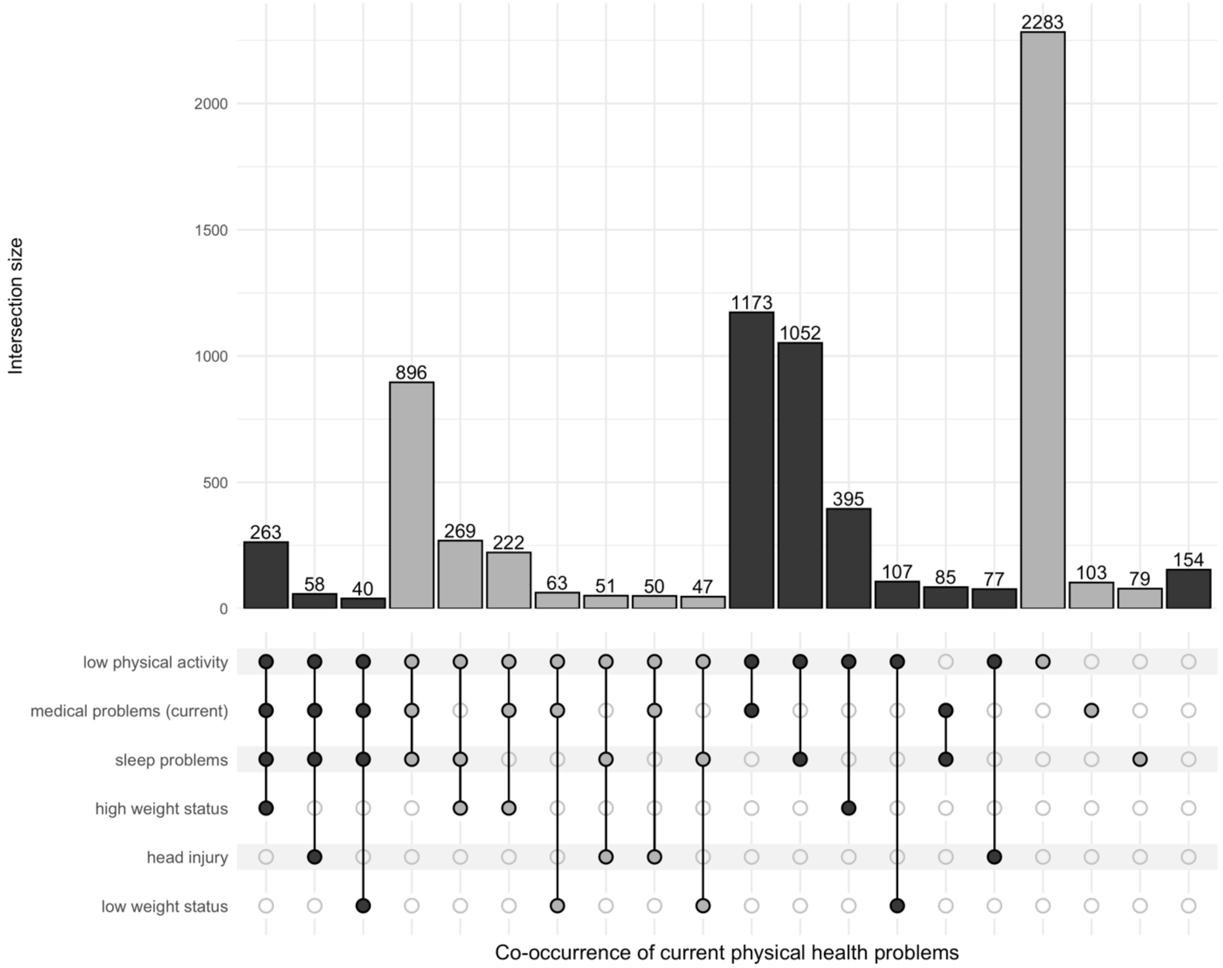

Supplementary Figure 3. Co-occurrence of current physical health problems. "UpSet" graph visualizing the co-occurrence of different lifetime/current physical health problems excluding perinatal complications. The dots in the matrix below the bar graph indicate a given combination of different problems. The bars above the dots indicate the number of youth exhibiting the respective combination. Only combinations of health problems that were reported by at least 30 participants are shown, therefore the sum of all the bars is lower than the overall sample size. For simplicity physical health factors have been collapsed to create a lower number of measures for overlap comparison. The most highly endorsed single current problem with no co-occurring problems was low physical activity (n=2283). Sleep problems co-occurred with several other factors: only 79 participants experienced sleep problems (including short sleep duration and high sleep disturbances) with no other co-occurring problem. The most common combination of current physical health problems only was low physical activity and lifetime medical problems (n=1173), followed by low physical activity and sleep problems (n=1052) and the co-occurrence of low physical activity, lifetime medical problems and sleep problems (n=896).

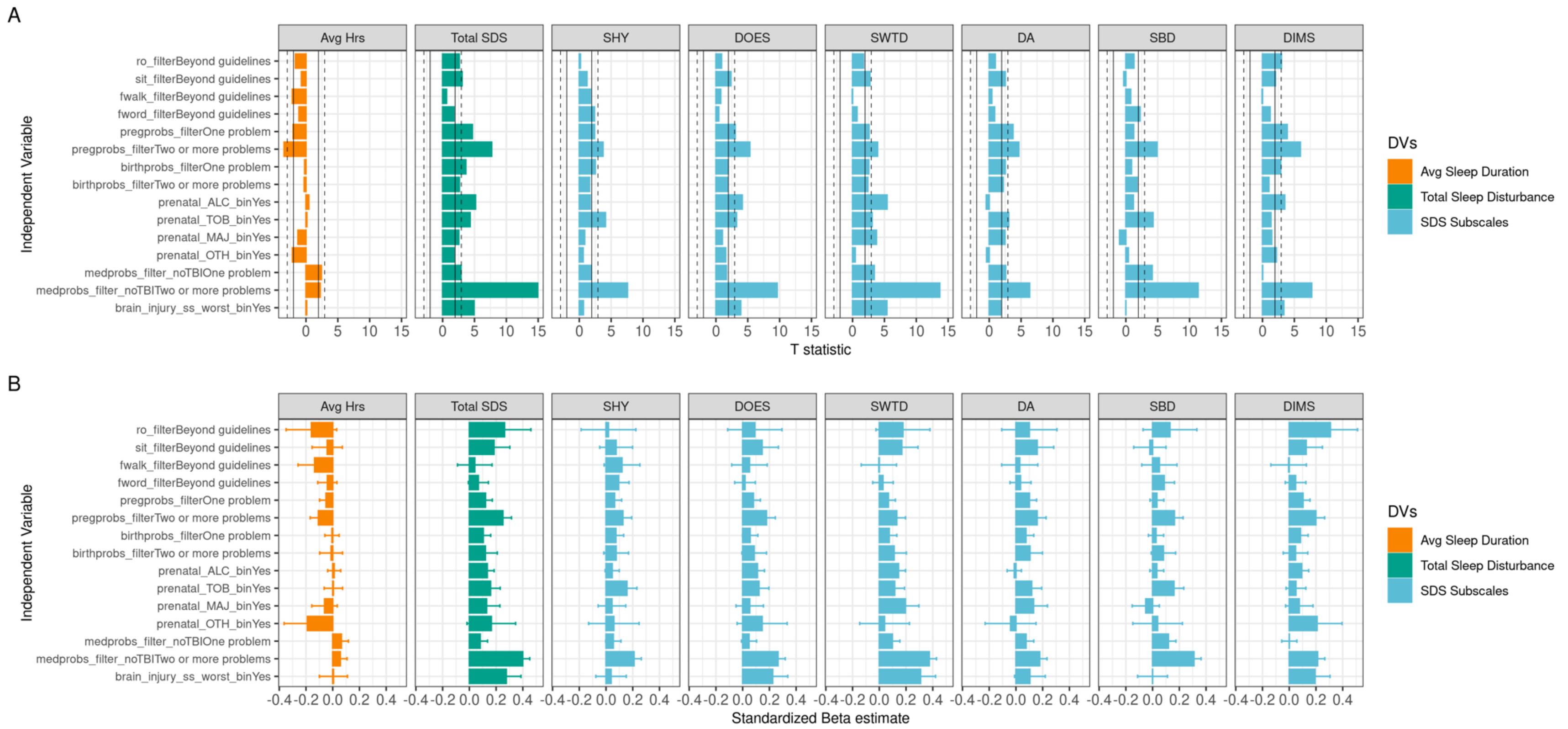

Supplementary Figure 4. Multiple regression statistics predicting sleep measures. Statistics for each physical health factor (y-axis) predicting each sleep measure (column) within a multiple regression model: A) t-statistic; B) standardized beta coefficients with 95% confidence intervals. The model also includes age, sex, pubertal development, race, ethnicity, household income and highest parental education for which the statistics are not shown. Physical health measures showed stronger associations with total sleep disturbance (green) compared to average hours sleep duration (orange). The pattern of associations were similar across sleep disturbance sub-scales (blue). Sleep-wake transition disorders (SWTD) and disorders of initiating and maintaining sleep (DIMS) showed the largest associations.

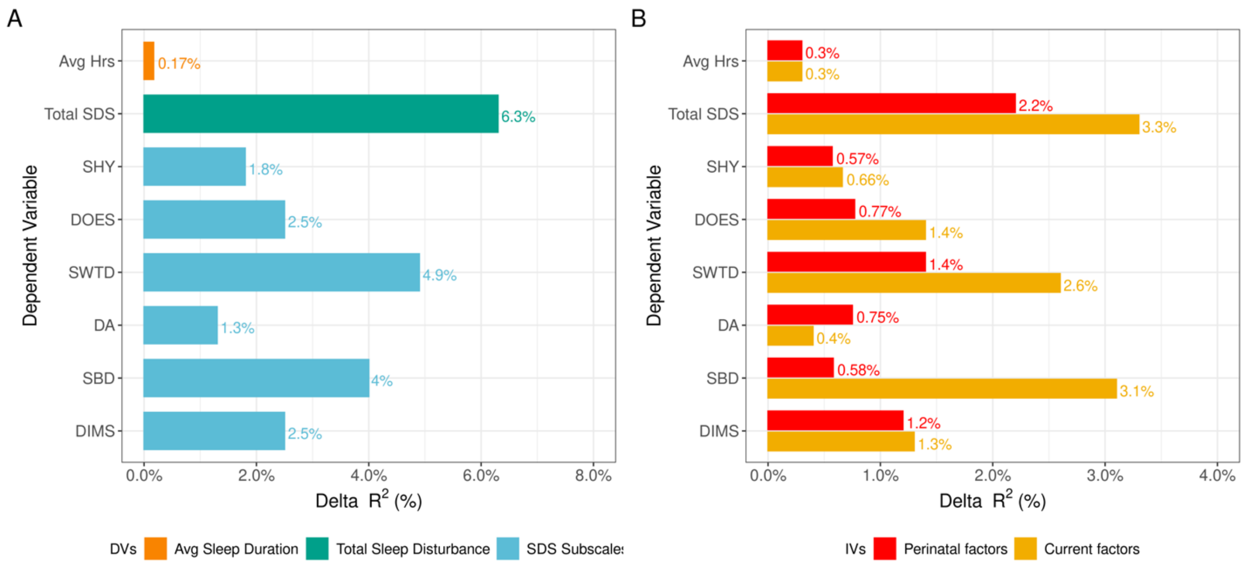

Supplementary Figure 5. Individual variability in sleep measures predicted by physical health factors including weight status. A) Out-of-sample cross-validated ΔR^2 estimates for each DV uniquely predicted by P and C together over and above covariates of no interest (age, PDS, assigned sex-at-birth, race, ethnicity, household income, parental education and data collection site; $|\text{Model 1} - \text{Model 2}|$). B) Out-of-sample cross-validated ΔR^2 estimates for each DV uniquely predicted by P (red; $|\text{Model 2} - \text{Model 3}|$) and C (yellow; $|\text{Model 2} - \text{Model 4}|$) controlling for covariates of no interest and the other IVs respectively. SDS sub-scales: sleep hyperhydrosis (SHY); disorders of excessive somnolence (DOES); sleep-wake transition disorders, (SWTD); disorders of arousal (DA); sleep breathing disorders, (SBD); disorders of initiating and maintaining sleep (DIMS).

A

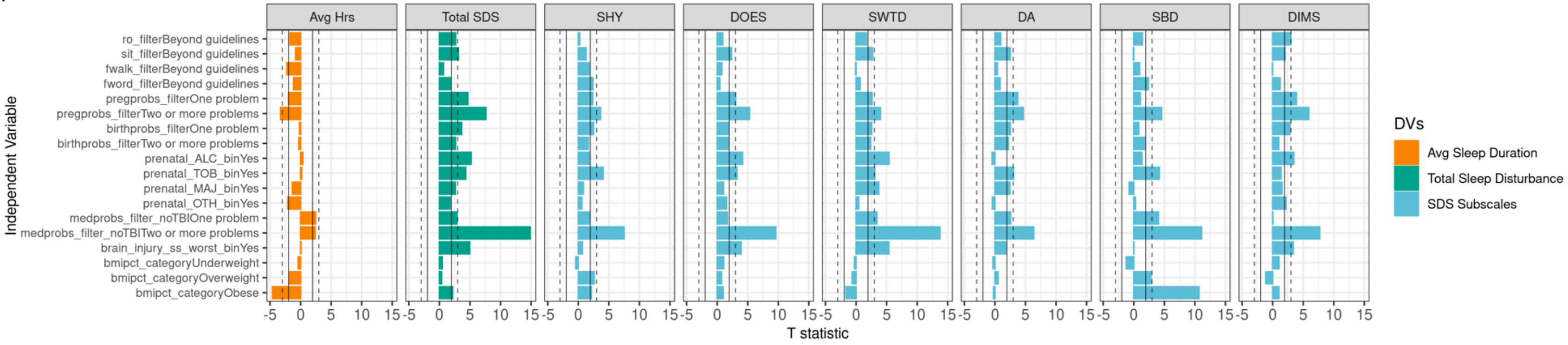

B

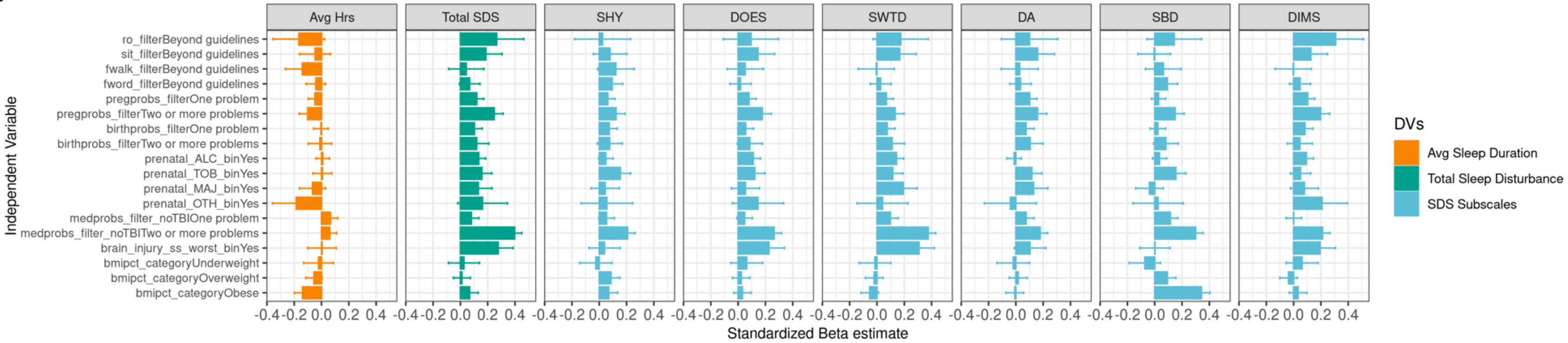

Supplementary Figure 6. Multiple regression statistics predicting sleep measures including weight status. Statistics for each physical health factor (y-axis) predicting each sleep measure (column) within a multiple regression model: A) t-statistic; B) standardized beta coefficients with 95% confidence intervals. The model also includes age, sex, pubertal development, race, ethnicity, household income and highest parental education for which the statistics are not shown. Having obesity showed a significant association with average hours sleep duration and sleep breathing disorders (SBD) as expected.

Supplementary Table 1. Odds ratios, 95% confidence intervals (CI) and p-values for the 458 logistic regression models completed for Figure 1.

DV	IV	Odds Ratio	Lower CI	Upper CI	PValue
**BMICategory_UW_Num**	BMICategory_Obese_Num	NA	NA	NA	NA
**BMICategory_UW_Num**	SportsActivitiesCat_Num	1.22	0.89	1.67	2.14E-01
**BMICategory_UW_Num**	SleepCat_Short_Num	0.98	0.66	1.46	9.26E-01
**BMICategory_UW_Num**	SleepDisturbanceCat_Num	0.98	0.76	1.28	8.97E-01
**BMICategory_UW_Num**	FirstWalkAgeCat_Num	1.35	0.73	2.52	3.41E-01
**BMICategory_UW_Num**	RollOverAgeCat_Num	0.86	0.27	2.74	7.94E-01
**BMICategory_UW_Num**	SittingAgeCat_Num	1.82	1.11	3.00	1.83E-02
**BMICategory_UW_Num**	FirstWordAgeCat_Num	1.36	0.94	1.96	1.04E-01
**BMICategory_UW_Num**	PrematurityCat_Num	1.33	0.92	1.92	1.28E-01
**BMICategory_UW_Num**	BirthweightCat_Low_Num	2.03	1.45	2.83	3.41E-05
**BMICategory_UW_Num**	BirthweightCat_High_Num	0.70	0.43	1.13	1.41E-01
**BMICategory_UW_Num**	MedProblemsDuringPregnancyCat_Num	0.96	0.68	1.37	8.38E-01
**BMICategory_UW_Num**	MedProblemsDuringBirthCat_Num	1.07	0.67	1.71	7.84E-01
**BMICategory_UW_Num**	PrenatalAlcoholCat_Num	0.93	0.71	1.23	6.28E-01
**BMICategory_UW_Num**	PrenatalTobaccoCat_Num	0.54	0.32	0.88	1.43E-02
**BMICategory_UW_Num**	PrenatalMarijuanaCat_Num	0.52	0.24	1.12	9.44E-02
**BMICategory_UW_Num**	PrenatalOtherSubstanceCat_Num	0.27	0.04	1.97	1.97E-01
**BMICategory_UW_Num**	PhysActivity_60MinVigorous_Num	1.12	0.81	1.55	5.05E-01
**BMICategory_UW_Num**	PhysActivity_Strengthening_Num	1.41	1.07	1.87	1.60E-02
**BMICategory_UW_Num**	LifeTimeMedProblemsCat_Num	1.00	0.78	1.28	9.85E-01
**BMICategory_UW_Num**	TBICat_Num	0.48	0.21	1.09	7.85E-02
**BMICategory_Obese_Num**	BMICategory_UW_Num	NA	NA	NA	NA
**BMICategory_Obese_Num**	SportsActivitiesCat_Num	1.22	1.05	1.42	8.17E-03
**BMICategory_Obese_Num**	SleepCat_Short_Num	1.28	1.08	1.51	3.49E-03
**BMICategory_Obese_Num**	SleepDisturbanceCat_Num	1.14	1.00	1.31	5.72E-02
**BMICategory_Obese_Num**	FirstWalkAgeCat_Num	0.63	0.42	0.96	3.03E-02
**BMICategory_Obese_Num**	RollOverAgeCat_Num	0.66	0.35	1.27	2.14E-01
**BMICategory_Obese_Num**	SittingAgeCat_Num	0.64	0.44	0.94	2.13E-02
**BMICategory_Obese_Num**	FirstWordAgeCat_Num	0.83	0.65	1.05	1.18E-01
**BMICategory_Obese_Num**	PrematurityCat_Num	1.08	0.87	1.34	4.87E-01
**BMICategory_Obese_Num**	BirthweightCat_Low_Num	0.86	0.70	1.06	1.58E-01
**BMICategory_Obese_Num**	BirthweightCat_High_Num	1.45	1.18	1.79	5.06E-04
**BMICategory_Obese_Num**	MedProblemsDuringPregnancyCat_Num	1.27	1.07	1.50	5.53E-03
**BMICategory_Obese_Num**	MedProblemsDuringBirthCat_Num	1.06	0.81	1.38	6.75E-01
**BMICategory_Obese_Num**	PrenatalAlcoholCat_Num	0.93	0.80	1.08	3.56E-01
**BMICategory_Obese_Num**	PrenatalTobaccoCat_Num	1.01	0.84	1.21	9.41E-01
**BMICategory_Obese_Num**	PrenatalMarijuanaCat_Num	0.75	0.57	0.97	2.83E-02
**BMICategory_Obese_Num**	PrenatalOtherSubstanceCat_Num	1.18	0.74	1.90	4.80E-01
**BMICategory_Obese_Num**	PhysActivity_60MinVigorous_Num	1.35	1.12	1.62	1.87E-03
**BMICategory_Obese_Num**	PhysActivity_Strengthening_Num	0.99	0.86	1.14	9.20E-01
**BMICategory_Obese_Num**	LifeTimeMedProblemsCat_Num	1.22	1.07	1.39	3.19E-03
**BMICategory_Obese_Num**	TBICat_Num	0.95	0.67	1.34	7.62E-01
**SportsActivitiesCat_Num**	BMICategory_UW_Num	1.23	0.89	1.68	2.06E-01
**SportsActivitiesCat_Num**	BMICategory_Obese_Num	1.23	1.06	1.43	6.20E-03
**SportsActivitiesCat_Num**	SleepCat_Short_Num	1.42	1.22	1.66	7.15E-06
**SportsActivitiesCat_Num**	SleepDisturbanceCat_Num	1.06	0.93	1.20	3.86E-01
**SportsActivitiesCat_Num**	FirstWalkAgeCat_Num	1.25	0.91	1.72	1.77E-01
**SportsActivitiesCat_Num**	RollOverAgeCat_Num	1.11	0.66	1.85	6.92E-01
**SportsActivitiesCat_Num**	SittingAgeCat_Num	1.14	0.84	1.53	4.00E-01
**SportsActivitiesCat_Num**	FirstWordAgeCat_Num	1.10	0.90	1.35	3.60E-01
**SportsActivitiesCat_Num**	PrematurityCat_Num	1.27	1.04	1.54	1.66E-02
**SportsActivitiesCat_Num**	BirthweightCat_Low_Num	1.19	0.99	1.43	6.59E-02
**SportsActivitiesCat_Num**	BirthweightCat_High_Num	1.03	0.83	1.26	8.11E-01
**SportsActivitiesCat_Num**	MedProblemsDuringPregnancyCat_Num	1.17	1.00	1.36	5.33E-02
**SportsActivitiesCat_Num**	MedProblemsDuringBirthCat_Num	1.11	0.87	1.41	4.07E-01
**SportsActivitiesCat_Num**	PrenatalAlcoholCat_Num	0.88	0.76	1.01	7.19E-02
**SportsActivitiesCat_Num**	PrenatalTobaccoCat_Num	1.00	0.85	1.19	9.61E-01
**SportsActivitiesCat_Num**	PrenatalMarijuanaCat_Num	1.02	0.81	1.28	8.83E-01
**SportsActivitiesCat_Num**	PrenatalOtherSubstanceCat_Num	0.87	0.55	1.37	5.43E-01
**SportsActivitiesCat_Num**	PhysActivity_60MinVigorous_Num	1.30	1.10	1.54	1.95E-03
**SportsActivitiesCat_Num**	PhysActivity_Strengthening_Num	1.22	1.07	1.39	2.41E-03

**SportsActivitiesCat_Num**	LifeTimeMedProblemsCat_Num	0.87	0.77	0.99	2.99E-02
**SportsActivitiesCat_Num**	TBICat_Num	0.90	0.65	1.24	5.15E-01
**SleepCat_Short_Num**	BMICategory_UW_Num	1.04	0.70	1.55	8.32E-01
**SleepCat_Short_Num**	BMICategory_Obese_Num	1.30	1.10	1.53	1.77E-03
**SleepCat_Short_Num**	SportsActivitiesCat_Num	1.43	1.23	1.67	5.12E-06
**SleepCat_Short_Num**	SleepDisturbanceCat_Num	3.19	2.76	3.67	2.82E-57
**SleepCat_Short_Num**	FirstWalkAgeCat_Num	1.77	1.26	2.47	8.79E-04
**SleepCat_Short_Num**	RollOverAgeCat_Num	1.52	0.87	2.66	1.38E-01
**SleepCat_Short_Num**	SittingAgeCat_Num	1.41	1.01	1.95	4.20E-02
**SleepCat_Short_Num**	FirstWordAgeCat_Num	1.25	0.99	1.57	6.26E-02
**SleepCat_Short_Num**	PrematurityCat_Num	0.98	0.78	1.24	8.59E-01
**SleepCat_Short_Num**	BirthweightCat_Low_Num	1.04	0.84	1.29	6.96E-01
**SleepCat_Short_Num**	BirthweightCat_High_Num	0.97	0.76	1.24	8.01E-01
**SleepCat_Short_Num**	MedProblemsDuringPregnancyCat_Num	1.26	1.06	1.51	9.46E-03
**SleepCat_Short_Num**	MedProblemsDuringBirthCat_Num	1.05	0.79	1.39	7.52E-01
**SleepCat_Short_Num**	PrenatalAlcoholCat_Num	1.07	0.91	1.26	3.88E-01
**SleepCat_Short_Num**	PrenatalTobaccoCat_Num	1.08	0.89	1.30	4.41E-01
**SleepCat_Short_Num**	PrenatalMarijuanaCat_Num	1.15	0.89	1.47	2.82E-01
**SleepCat_Short_Num**	PrenatalOtherSubstanceCat_Num	1.80	1.14	2.84	1.12E-02
**SleepCat_Short_Num**	PhysActivity_60MinVigorous_Num	1.02	0.84	1.23	8.55E-01
**SleepCat_Short_Num**	PhysActivity_Strengthening_Num	0.97	0.84	1.13	7.29E-01
**SleepCat_Short_Num**	LifeTimeMedProblemsCat_Num	1.00	0.87	1.16	9.48E-01
**SleepCat_Short_Num**	TBICat_Num	1.19	0.84	1.68	3.41E-01
**SleepDisturbanceCat_Num**	BMICategory_UW_Num	1.04	0.80	1.36	7.46E-01
**SleepDisturbanceCat_Num**	BMICategory_Obese_Num	1.13	0.99	1.30	6.87E-02
**SleepDisturbanceCat_Num**	SportsActivitiesCat_Num	1.07	0.94	1.21	3.10E-01
**SleepDisturbanceCat_Num**	SleepCat_Short_Num	3.14	2.72	3.62	2.39E-56
**SleepDisturbanceCat_Num**	FirstWalkAgeCat_Num	1.25	0.95	1.64	1.16E-01
**SleepDisturbanceCat_Num**	RollOverAgeCat_Num	1.52	0.99	2.34	5.69E-02
**SleepDisturbanceCat_Num**	SittingAgeCat_Num	1.59	1.24	2.03	2.05E-04
**SleepDisturbanceCat_Num**	FirstWordAgeCat_Num	1.24	1.05	1.47	9.96E-03
**SleepDisturbanceCat_Num**	PrematurityCat_Num	0.95	0.80	1.13	5.78E-01
**SleepDisturbanceCat_Num**	BirthweightCat_Low_Num	0.96	0.81	1.13	5.88E-01
**SleepDisturbanceCat_Num**	BirthweightCat_High_Num	0.95	0.80	1.13	5.95E-01
**SleepDisturbanceCat_Num**	MedProblemsDuringPregnancyCat_Num	1.63	1.42	1.86	1.31E-12
**SleepDisturbanceCat_Num**	MedProblemsDuringBirthCat_Num	1.30	1.07	1.59	8.56E-03
**SleepDisturbanceCat_Num**	PrenatalAlcoholCat_Num	1.46	1.30	1.63	3.41E-11
**SleepDisturbanceCat_Num**	PrenatalTobaccoCat_Num	1.47	1.27	1.70	3.61E-07
**SleepDisturbanceCat_Num**	PrenatalMarijuanaCat_Num	1.62	1.32	1.98	3.21E-06
**SleepDisturbanceCat_Num**	PrenatalOtherSubstanceCat_Num	1.56	1.06	2.29	2.55E-02
**SleepDisturbanceCat_Num**	PhysActivity_60MinVigorous_Num	0.94	0.82	1.07	3.37E-01
**SleepDisturbanceCat_Num**	PhysActivity_Strengthening_Num	0.98	0.88	1.09	7.26E-01
**SleepDisturbanceCat_Num**	LifeTimeMedProblemsCat_Num	2.08	1.88	2.31	3.48E-46
**SleepDisturbanceCat_Num**	TBICat_Num	1.84	1.45	2.34	5.45E-07
**FirstWalkAgeCat_Num**	BMICategory_UW_Num	1.36	0.73	2.53	3.39E-01
**FirstWalkAgeCat_Num**	BMICategory_Obese_Num	0.61	0.41	0.93	2.03E-02
**FirstWalkAgeCat_Num**	SportsActivitiesCat_Num	1.24	0.90	1.70	1.88E-01
**FirstWalkAgeCat_Num**	SleepCat_Short_Num	1.73	1.24	2.43	1.34E-03
**FirstWalkAgeCat_Num**	SleepDisturbanceCat_Num	1.24	0.94	1.63	1.25E-01
**FirstWalkAgeCat_Num**	RollOverAgeCat_Num	4.08	2.10	7.93	3.31E-05
**FirstWalkAgeCat_Num**	SittingAgeCat_Num	18.27	13.34	25.02	2.36E-73
**FirstWalkAgeCat_Num**	FirstWordAgeCat_Num	5.04	3.77	6.75	1.61E-27
**FirstWalkAgeCat_Num**	PrematurityCat_Num	2.53	1.79	3.57	1.37E-07
**FirstWalkAgeCat_Num**	BirthweightCat_Low_Num	2.69	1.91	3.78	1.53E-08
**FirstWalkAgeCat_Num**	BirthweightCat_High_Num	0.58	0.33	1.01	5.32E-02
**FirstWalkAgeCat_Num**	MedProblemsDuringPregnancyCat_Num	1.47	1.06	2.05	2.12E-02
**FirstWalkAgeCat_Num**	MedProblemsDuringBirthCat_Num	2.20	1.46	3.29	1.40E-04
**FirstWalkAgeCat_Num**	PrenatalAlcoholCat_Num	1.17	0.87	1.57	3.01E-01
**FirstWalkAgeCat_Num**	PrenatalTobaccoCat_Num	1.25	0.85	1.83	2.50E-01
**FirstWalkAgeCat_Num**	PrenatalMarijuanaCat_Num	1.36	0.82	2.27	2.35E-01
**FirstWalkAgeCat_Num**	PrenatalOtherSubstanceCat_Num	1.43	0.57	3.59	4.43E-01
**FirstWalkAgeCat_Num**	PhysActivity_60MinVigorous_Num	1.16	0.81	1.67	4.12E-01
**FirstWalkAgeCat_Num**	PhysActivity_Strengthening_Num	1.13	0.85	1.51	3.95E-01
**FirstWalkAgeCat_Num**	LifeTimeMedProblemsCat_Num	1.23	0.95	1.61	1.19E-01
**FirstWalkAgeCat_Num**	TBICat_Num	1.24	0.68	2.25	4.85E-01
**RollOverAgeCat_Num**	BMICategory_UW_Num	0.87	0.27	2.78	8.10E-01

**RollOverAgeCat_Num**	BMICategory_Obese_Num	0.68	0.36	1.28	2.27E-01
**RollOverAgeCat_Num**	SportsActivitiesCat_Num	1.06	0.64	1.77	8.18E-01
**RollOverAgeCat_Num**	SleepCat_Short_Num	1.48	0.85	2.58	1.62E-01
**RollOverAgeCat_Num**	SleepDisturbanceCat_Num	1.49	0.96	2.29	7.22E-02
**RollOverAgeCat_Num**	FirstWalkAgeCat_Num	4.14	2.14	8.03	2.57E-05
**RollOverAgeCat_Num**	SittingAgeCat_Num	12.19	7.57	19.62	7.32E-25
**RollOverAgeCat_Num**	FirstWordAgeCat_Num	4.05	2.52	6.50	7.08E-09
**RollOverAgeCat_Num**	PrematurityCat_Num	3.57	2.18	5.86	4.61E-07
**RollOverAgeCat_Num**	BirthweightCat_Low_Num	2.02	1.16	3.49	1.23E-02
**RollOverAgeCat_Num**	BirthweightCat_High_Num	1.23	0.63	2.41	5.40E-01
**RollOverAgeCat_Num**	MedProblemsDuringPregnancyCat_Num	1.67	1.01	2.76	4.43E-02
**RollOverAgeCat_Num**	MedProblemsDuringBirthCat_Num	1.54	0.76	3.13	2.34E-01
**RollOverAgeCat_Num**	PrenatalAlcoholCat_Num	0.89	0.54	1.46	6.35E-01
**RollOverAgeCat_Num**	PrenatalTobaccoCat_Num	0.86	0.44	1.67	6.56E-01
**RollOverAgeCat_Num**	PrenatalMarijuanaCat_Num	0.84	0.30	2.35	7.43E-01
**RollOverAgeCat_Num**	PrenatalOtherSubstanceCat_Num	1.60	0.38	6.70	5.17E-01
**RollOverAgeCat_Num**	PhysActivity_60MinVigorous_Num	1.33	0.72	2.46	3.65E-01
**RollOverAgeCat_Num**	PhysActivity_Strengthening_Num	0.94	0.60	1.47	7.97E-01
**RollOverAgeCat_Num**	LifeTimeMedProblemsCat_Num	1.26	0.83	1.92	2.77E-01
**RollOverAgeCat_Num**	TBICat_Num	1.22	0.44	3.38	6.99E-01
**SittingAgeCat_Num**	BMICategory_UW_Num	1.81	1.10	2.98	1.93E-02
**SittingAgeCat_Num**	BMICategory_Obese_Num	0.59	0.40	0.86	6.65E-03
**SittingAgeCat_Num**	SportsActivitiesCat_Num	1.10	0.82	1.49	5.16E-01
**SittingAgeCat_Num**	SleepCat_Short_Num	1.37	0.99	1.90	6.10E-02
**SittingAgeCat_Num**	SleepDisturbanceCat_Num	1.55	1.21	1.98	4.67E-04
**SittingAgeCat_Num**	FirstWalkAgeCat_Num	18.30	13.37	25.05	9.57E-74
**SittingAgeCat_Num**	RollOverAgeCat_Num	12.10	7.52	19.49	1.02E-24
**SittingAgeCat_Num**	FirstWordAgeCat_Num	5.72	4.41	7.42	2.33E-39
**SittingAgeCat_Num**	PrematurityCat_Num	3.77	2.84	5.01	5.61E-20
**SittingAgeCat_Num**	BirthweightCat_Low_Num	4.02	3.01	5.36	3.05E-21
**SittingAgeCat_Num**	BirthweightCat_High_Num	0.78	0.50	1.22	2.79E-01
**SittingAgeCat_Num**	MedProblemsDuringPregnancyCat_Num	2.14	1.62	2.81	6.01E-08
**SittingAgeCat_Num**	MedProblemsDuringBirthCat_Num	1.75	1.19	2.57	4.08E-03
**SittingAgeCat_Num**	PrenatalAlcoholCat_Num	0.85	0.65	1.13	2.65E-01
**SittingAgeCat_Num**	PrenatalTobaccoCat_Num	1.24	0.88	1.76	2.15E-01
**SittingAgeCat_Num**	PrenatalMarijuanaCat_Num	0.63	0.34	1.18	1.51E-01
**SittingAgeCat_Num**	PrenatalOtherSubstanceCat_Num	1.74	0.79	3.81	1.69E-01
**SittingAgeCat_Num**	PhysActivity_60MinVigorous_Num	0.99	0.72	1.35	9.28E-01
**SittingAgeCat_Num**	PhysActivity_Strengthening_Num	1.01	0.78	1.30	9.47E-01
**SittingAgeCat_Num**	LifeTimeMedProblemsCat_Num	1.51	1.19	1.92	6.25E-04
**SittingAgeCat_Num**	TBICat_Num	1.63	0.99	2.68	5.38E-02
**FirstWordAgeCat_Num**	BMICategory_UW_Num	1.35	0.94	1.95	1.07E-01
**FirstWordAgeCat_Num**	BMICategory_Obese_Num	0.79	0.62	1.00	5.20E-02
**FirstWordAgeCat_Num**	SportsActivitiesCat_Num	1.09	0.89	1.34	3.93E-01
**FirstWordAgeCat_Num**	SleepCat_Short_Num	1.22	0.97	1.54	8.45E-02
**FirstWordAgeCat_Num**	SleepDisturbanceCat_Num	1.22	1.03	1.44	2.10E-02
**FirstWordAgeCat_Num**	FirstWalkAgeCat_Num	5.04	3.76	6.75	2.00E-27
**FirstWordAgeCat_Num**	RollOverAgeCat_Num	4.04	2.52	6.49	7.22E-09
**FirstWordAgeCat_Num**	SittingAgeCat_Num	5.74	4.43	7.45	1.36E-39
**FirstWordAgeCat_Num**	PrematurityCat_Num	1.85	1.48	2.31	7.04E-08
**FirstWordAgeCat_Num**	BirthweightCat_Low_Num	2.04	1.64	2.55	2.89E-10
**FirstWordAgeCat_Num**	BirthweightCat_High_Num	0.88	0.67	1.16	3.69E-01
**FirstWordAgeCat_Num**	MedProblemsDuringPregnancyCat_Num	1.58	1.29	1.94	8.13E-06
**FirstWordAgeCat_Num**	MedProblemsDuringBirthCat_Num	1.66	1.27	2.18	2.47E-04
**FirstWordAgeCat_Num**	PrenatalAlcoholCat_Num	0.89	0.75	1.07	2.16E-01
**FirstWordAgeCat_Num**	PrenatalTobaccoCat_Num	1.05	0.82	1.35	6.89E-01
**FirstWordAgeCat_Num**	PrenatalMarijuanaCat_Num	1.00	0.70	1.43	9.96E-01
**FirstWordAgeCat_Num**	PrenatalOtherSubstanceCat_Num	0.93	0.47	1.86	8.41E-01
**FirstWordAgeCat_Num**	PhysActivity_60MinVigorous_Num	1.07	0.87	1.33	5.03E-01
**FirstWordAgeCat_Num**	PhysActivity_Strengthening_Num	1.24	1.04	1.48	1.50E-02
**FirstWordAgeCat_Num**	LifeTimeMedProblemsCat_Num	1.30	1.11	1.53	9.94E-04
**FirstWordAgeCat_Num**	TBICat_Num	1.10	0.76	1.61	6.09E-01
**PrematurityCat_Num**	BMICategory_UW_Num	1.33	0.92	1.93	1.24E-01
**PrematurityCat_Num**	BMICategory_Obese_Num	1.08	0.87	1.33	4.87E-01
**PrematurityCat_Num**	SportsActivitiesCat_Num	1.26	1.04	1.52	2.08E-02
**PrematurityCat_Num**	SleepCat_Short_Num	0.97	0.77	1.22	7.85E-01

**PrematurityCat_Num**	SleepDisturbanceCat_Num	0.95	0.80	1.13	5.85E-01
**PrematurityCat_Num**	FirstWalkAgeCat_Num	2.52	1.78	3.55	1.45E-07
**PrematurityCat_Num**	RollOverAgeCat_Num	3.54	2.16	5.82	5.58E-07
**PrematurityCat_Num**	SittingAgeCat_Num	3.76	2.83	4.99	4.38E-20
**PrematurityCat_Num**	FirstWordAgeCat_Num	1.85	1.48	2.31	7.52E-08
**PrematurityCat_Num**	BirthweightCat_Low_Num	26.26	21.61	31.91	#########
**PrematurityCat_Num**	BirthweightCat_High_Num	0.06	0.03	0.15	9.46E-10
**PrematurityCat_Num**	MedProblemsDuringPregnancyCat_Num	3.65	3.07	4.36	2.07E-47
**PrematurityCat_Num**	MedProblemsDuringBirthCat_Num	5.73	4.58	7.16	5.43E-53
**PrematurityCat_Num**	PrenatalAlcoholCat_Num	0.75	0.63	0.90	1.98E-03
**PrematurityCat_Num**	PrenatalTobaccoCat_Num	1.19	0.95	1.49	1.32E-01
**PrematurityCat_Num**	PrenatalMarijuanaCat_Num	0.94	0.66	1.33	7.19E-01
**PrematurityCat_Num**	PrenatalOtherSubstanceCat_Num	0.99	0.53	1.87	9.84E-01
**PrematurityCat_Num**	PhysActivity_60MinVigorous_Num	0.81	0.67	0.99	3.72E-02
**PrematurityCat_Num**	PhysActivity_Strengthening_Num	1.05	0.89	1.24	5.59E-01
**PrematurityCat_Num**	LifeTimeMedProblemsCat_Num	1.18	1.01	1.38	3.55E-02
**PrematurityCat_Num**	TBICat_Num	1.12	0.77	1.64	5.47E-01
**BirthweightCat_Low_Num**	BMICategory_UW_Num	2.02	1.44	2.81	3.75E-05
**BirthweightCat_Low_Num**	BMICategory_Obese_Num	0.87	0.70	1.07	1.87E-01
**BirthweightCat_Low_Num**	SportsActivitiesCat_Num	1.18	0.98	1.42	7.76E-02
**BirthweightCat_Low_Num**	SleepCat_Short_Num	1.04	0.84	1.29	7.14E-01
**BirthweightCat_Low_Num**	SleepDisturbanceCat_Num	0.96	0.82	1.14	6.62E-01
**BirthweightCat_Low_Num**	FirstWalkAgeCat_Num	2.67	1.90	3.76	1.36E-08
**BirthweightCat_Low_Num**	RollOverAgeCat_Num	1.98	1.14	3.43	1.51E-02
**BirthweightCat_Low_Num**	SittingAgeCat_Num	3.98	2.99	5.31	3.40E-21
**BirthweightCat_Low_Num**	FirstWordAgeCat_Num	2.06	1.65	2.57	1.61E-10
**BirthweightCat_Low_Num**	PrematurityCat_Num	26.27	21.63	31.89	#########
**BirthweightCat_Low_Num**	BirthweightCat_High_Num	NA	NA	NA	NA
**BirthweightCat_Low_Num**	MedProblemsDuringPregnancyCat_Num	3.08	2.59	3.67	1.32E-36
**BirthweightCat_Low_Num**	MedProblemsDuringBirthCat_Num	4.43	3.52	5.57	3.76E-37
**BirthweightCat_Low_Num**	PrenatalAlcoholCat_Num	0.90	0.75	1.07	2.17E-01
**BirthweightCat_Low_Num**	PrenatalTobaccoCat_Num	1.35	1.09	1.67	6.24E-03
**BirthweightCat_Low_Num**	PrenatalMarijuanaCat_Num	1.05	0.76	1.44	7.63E-01
**BirthweightCat_Low_Num**	PrenatalOtherSubstanceCat_Num	1.03	0.56	1.88	9.33E-01
**BirthweightCat_Low_Num**	PhysActivity_60MinVigorous_Num	0.98	0.81	1.20	8.69E-01
**BirthweightCat_Low_Num**	PhysActivity_Strengthening_Num	1.01	0.86	1.18	9.31E-01
**BirthweightCat_Low_Num**	LifeTimeMedProblemsCat_Num	1.09	0.94	1.27	2.57E-01
**BirthweightCat_Low_Num**	TBICat_Num	1.09	0.74	1.59	6.74E-01
**BirthweightCat_High_Num**	BMICategory_UW_Num	0.70	0.43	1.12	1.36E-01
**BirthweightCat_High_Num**	BMICategory_Obese_Num	1.45	1.18	1.78	4.99E-04
**BirthweightCat_High_Num**	SportsActivitiesCat_Num	1.02	0.83	1.25	8.71E-01
**BirthweightCat_High_Num**	SleepCat_Short_Num	0.96	0.75	1.23	7.36E-01
**BirthweightCat_High_Num**	SleepDisturbanceCat_Num	0.95	0.80	1.13	5.96E-01
**BirthweightCat_High_Num**	FirstWalkAgeCat_Num	0.59	0.34	1.02	6.00E-02
**BirthweightCat_High_Num**	RollOverAgeCat_Num	1.23	0.63	2.40	5.53E-01
**BirthweightCat_High_Num**	SittingAgeCat_Num	0.78	0.50	1.21	2.68E-01
**BirthweightCat_High_Num**	FirstWordAgeCat_Num	0.89	0.68	1.17	4.12E-01
**BirthweightCat_High_Num**	PrematurityCat_Num	0.06	0.03	0.15	8.86E-10
**BirthweightCat_High_Num**	BirthweightCat_Low_Num	NA	NA	NA	NA
**BirthweightCat_High_Num**	MedProblemsDuringPregnancyCat_Num	0.79	0.61	1.01	5.54E-02
**BirthweightCat_High_Num**	MedProblemsDuringBirthCat_Num	0.92	0.66	1.29	6.33E-01
**BirthweightCat_High_Num**	PrenatalAlcoholCat_Num	0.85	0.71	1.03	9.06E-02
**BirthweightCat_High_Num**	PrenatalTobaccoCat_Num	0.65	0.49	0.86	2.78E-03
**BirthweightCat_High_Num**	PrenatalMarijuanaCat_Num	0.90	0.62	1.30	5.68E-01
**BirthweightCat_High_Num**	PrenatalOtherSubstanceCat_Num	0.58	0.25	1.33	1.97E-01
**BirthweightCat_High_Num**	PhysActivity_60MinVigorous_Num	1.13	0.91	1.41	2.58E-01
**BirthweightCat_High_Num**	PhysActivity_Strengthening_Num	0.97	0.82	1.15	7.38E-01
**BirthweightCat_High_Num**	LifeTimeMedProblemsCat_Num	1.03	0.88	1.21	6.84E-01
**BirthweightCat_High_Num**	TBICat_Num	1.11	0.76	1.63	5.76E-01
**MedProblemsDuringPregnancyCat_Num**	BMICategory_UW_Num	0.96	0.68	1.37	8.38E-01
**MedProblemsDuringPregnancyCat_Num**	BMICategory_Obese_Num	1.33	1.13	1.56	7.67E-04
**MedProblemsDuringPregnancyCat_Num**	SportsActivitiesCat_Num	1.17	1.00	1.37	4.65E-02

**MedProblemsDuringPregnancyCat_Num**	SleepCat_Short_Num	1.27	1.07	1.51	7.48E-03
**MedProblemsDuringPregnancyCat_Num**	SleepDisturbanceCat_Num	1.65	1.45	1.89	1.62E-13
**MedProblemsDuringPregnancyCat_Num**	FirstWalkAgeCat_Num	1.47	1.06	2.05	2.16E-02
**MedProblemsDuringPregnancyCat_Num**	RollOverAgeCat_Num	1.64	0.99	2.71	5.28E-02
**MedProblemsDuringPregnancyCat_Num**	SittingAgeCat_Num	2.14	1.62	2.82	6.01E-08
**MedProblemsDuringPregnancyCat_Num**	FirstWordAgeCat_Num	1.58	1.29	1.93	1.01E-05
**MedProblemsDuringPregnancyCat_Num**	PrematurityCat_Num	3.67	3.08	4.37	1.56E-47
**MedProblemsDuringPregnancyCat_Num**	BirthweightCat_Low_Num	3.09	2.59	3.68	1.62E-36
**MedProblemsDuringPregnancyCat_Num**	BirthweightCat_High_Num	0.78	0.61	1.00	5.30E-02
**MedProblemsDuringPregnancyCat_Num**	MedProblemsDuringBirthCat_Num	2.31	1.86	2.87	3.77E-14
**MedProblemsDuringPregnancyCat_Num**	PrenatalAlcoholCat_Num	1.05	0.91	1.22	4.95E-01
**MedProblemsDuringPregnancyCat_Num**	PrenatalTobaccoCat_Num	1.13	0.94	1.36	2.01E-01
**MedProblemsDuringPregnancyCat_Num**	PrenatalMarijuanaCat_Num	1.23	0.95	1.58	1.12E-01
**MedProblemsDuringPregnancyCat_Num**	PrenatalOtherSubstanceCat_Num	1.37	0.86	2.19	1.83E-01
**MedProblemsDuringPregnancyCat_Num**	PhysActivity_60MinVigorous_Num	1.01	0.85	1.21	8.82E-01
**MedProblemsDuringPregnancyCat_Num**	PhysActivity_Strengthening_Num	1.20	1.04	1.38	1.24E-02
**MedProblemsDuringPregnancyCat_Num**	LifeTimeMedProblemsCat_Num	1.69	1.48	1.93	2.72E-15
**MedProblemsDuringPregnancyCat_Num**	TBICat_Num	1.55	1.15	2.09	4.03E-03
**MedProblemsDuringBirthCat_Num**	BMICategory_UW_Num	1.06	0.66	1.70	8.09E-01
**MedProblemsDuringBirthCat_Num**	BMICategory_Obese_Num	1.05	0.81	1.37	7.06E-01
**MedProblemsDuringBirthCat_Num**	SportsActivitiesCat_Num	1.09	0.85	1.38	4.96E-01
**MedProblemsDuringBirthCat_Num**	SleepCat_Short_Num	1.03	0.77	1.36	8.61E-01
**MedProblemsDuringBirthCat_Num**	SleepDisturbanceCat_Num	1.31	1.07	1.59	7.89E-03
**MedProblemsDuringBirthCat_Num**	FirstWalkAgeCat_Num	2.21	1.48	3.32	1.19E-04
**MedProblemsDuringBirthCat_Num**	RollOverAgeCat_Num	1.53	0.75	3.10	2.44E-01
**MedProblemsDuringBirthCat_Num**	SittingAgeCat_Num	1.76	1.20	2.57	3.87E-03
**MedProblemsDuringBirthCat_Num**	FirstWordAgeCat_Num	1.66	1.26	2.18	2.65E-04
**MedProblemsDuringBirthCat_Num**	PrematurityCat_Num	5.78	4.62	7.22	2.35E-53
**MedProblemsDuringBirthCat_Num**	BirthweightCat_Low_Num	4.44	3.52	5.58	7.14E-37
**MedProblemsDuringBirthCat_Num**	BirthweightCat_High_Num	0.92	0.65	1.29	6.14E-01
**MedProblemsDuringBirthCat_Num**	MedProblemsDuringPregnancyCat_Num	2.31	1.86	2.87	3.72E-14
**MedProblemsDuringBirthCat_Num**	PrenatalAlcoholCat_Num	0.95	0.77	1.18	6.65E-01
**MedProblemsDuringBirthCat_Num**	PrenatalTobaccoCat_Num	1.45	1.11	1.89	6.10E-03
**MedProblemsDuringBirthCat_Num**	PrenatalMarijuanaCat_Num	0.96	0.63	1.46	8.34E-01
**MedProblemsDuringBirthCat_Num**	PrenatalOtherSubstanceCat_Num	0.68	0.27	1.70	4.10E-01
**MedProblemsDuringBirthCat_Num**	PhysActivity_60MinVigorous_Num	0.85	0.67	1.08	1.76E-01
**MedProblemsDuringBirthCat_Num**	PhysActivity_Strengthening_Num	0.95	0.78	1.16	6.10E-01
**MedProblemsDuringBirthCat_Num**	LifeTimeMedProblemsCat_Num	1.40	1.16	1.69	4.34E-04
**MedProblemsDuringBirthCat_Num**	TBICat_Num	1.85	1.26	2.72	1.85E-03
**PrenatalAlcoholCat_Num**	BMICategory_UW_Num	0.93	0.71	1.23	6.22E-01
**PrenatalAlcoholCat_Num**	BMICategory_Obese_Num	0.95	0.81	1.10	4.74E-01
**PrenatalAlcoholCat_Num**	SportsActivitiesCat_Num	0.89	0.78	1.03	1.19E-01
**PrenatalAlcoholCat_Num**	SleepCat_Short_Num	1.09	0.93	1.28	3.05E-01
**PrenatalAlcoholCat_Num**	SleepDisturbanceCat_Num	1.47	1.31	1.64	1.23E-11
**PrenatalAlcoholCat_Num**	FirstWalkAgeCat_Num	1.17	0.87	1.57	2.99E-01
**PrenatalAlcoholCat_Num**	RollOverAgeCat_Num	0.90	0.55	1.48	6.73E-01
**PrenatalAlcoholCat_Num**	SittingAgeCat_Num	0.86	0.65	1.13	2.82E-01
**PrenatalAlcoholCat_Num**	FirstWordAgeCat_Num	0.90	0.75	1.07	2.31E-01
**PrenatalAlcoholCat_Num**	PrematurityCat_Num	0.76	0.63	0.91	2.50E-03

**PrenatalAlcoholCat_Num**	BirthweightCat_Low_Num	0.90	0.76	1.07	2.29E-01
**PrenatalAlcoholCat_Num**	BirthweightCat_High_Num	0.86	0.72	1.03	9.96E-02
**PrenatalAlcoholCat_Num**	MedProblemsDuringPregnancyCat_Num	1.06	0.91	1.23	4.29E-01
**PrenatalAlcoholCat_Num**	MedProblemsDuringBirthCat_Num	0.96	0.78	1.19	7.20E-01
**PrenatalAlcoholCat_Num**	PrenatalTobaccoCat_Num	5.20	4.42	6.12	4.85E-88
**PrenatalAlcoholCat_Num**	PrenatalMarijuanaCat_Num	7.79	6.22	9.75	2.02E-71
**PrenatalAlcoholCat_Num**	PrenatalOtherSubstanceCat_Num	5.30	3.53	7.97	1.01E-15
**PrenatalAlcoholCat_Num**	PhysActivity_60MinVigorous_Num	0.96	0.83	1.10	5.32E-01
**PrenatalAlcoholCat_Num**	PhysActivity_Strengthening_Num	1.06	0.95	1.19	2.92E-01
**PrenatalAlcoholCat_Num**	LifeTimeMedProblemsCat_Num	1.09	0.98	1.21	1.27E-01
**PrenatalAlcoholCat_Num**	TBICat_Num	0.99	0.76	1.29	9.38E-01
**PrenatalTobaccoCat_Num**	BMICategory_UW_Num	0.52	0.31	0.86	1.12E-02
**PrenatalTobaccoCat_Num**	BMICategory_Obese_Num	1.05	0.88	1.26	5.62E-01
**PrenatalTobaccoCat_Num**	SportsActivitiesCat_Num	0.98	0.83	1.16	7.94E-01
**PrenatalTobaccoCat_Num**	SleepCat_Short_Num	1.05	0.87	1.27	6.13E-01
**PrenatalTobaccoCat_Num**	SleepDisturbanceCat_Num	1.50	1.29	1.73	9.89E-08
**PrenatalTobaccoCat_Num**	FirstWalkAgeCat_Num	1.22	0.83	1.80	3.10E-01
**PrenatalTobaccoCat_Num**	RollOverAgeCat_Num	0.83	0.43	1.63	5.91E-01
**PrenatalTobaccoCat_Num**	SittingAgeCat_Num	1.22	0.87	1.73	2.51E-01
**PrenatalTobaccoCat_Num**	FirstWordAgeCat_Num	1.04	0.81	1.34	7.36E-01
**PrenatalTobaccoCat_Num**	PrematurityCat_Num	1.20	0.96	1.51	1.15E-01
**PrenatalTobaccoCat_Num**	BirthweightCat_Low_Num	1.34	1.08	1.66	7.46E-03
**PrenatalTobaccoCat_Num**	BirthweightCat_High_Num	0.67	0.51	0.89	5.25E-03
**PrenatalTobaccoCat_Num**	MedProblemsDuringPregnancyCat_Num	1.11	0.92	1.34	2.56E-01
**PrenatalTobaccoCat_Num**	MedProblemsDuringBirthCat_Num	1.43	1.09	1.86	8.65E-03
**PrenatalTobaccoCat_Num**	PrenatalAlcoholCat_Num	5.19	4.41	6.11	2.61E-87
**PrenatalTobaccoCat_Num**	PrenatalMarijuanaCat_Num	9.74	7.75	12.25	1.63E-84
**PrenatalTobaccoCat_Num**	PrenatalOtherSubstanceCat_Num	7.53	4.88	11.64	8.83E-20
**PrenatalTobaccoCat_Num**	PhysActivity_60MinVigorous_Num	0.91	0.76	1.11	3.57E-01
**PrenatalTobaccoCat_Num**	PhysActivity_Strengthening_Num	0.97	0.83	1.13	6.64E-01
**PrenatalTobaccoCat_Num**	LifeTimeMedProblemsCat_Num	1.17	1.01	1.36	3.28E-02
**PrenatalTobaccoCat_Num**	TBICat_Num	1.40	0.98	1.98	6.36E-02
**PrenatalMarijuanaCat_Num**	BMICategory_UW_Num	0.51	0.24	1.09	8.36E-02
**PrenatalMarijuanaCat_Num**	BMICategory_Obese_Num	0.78	0.60	1.01	5.77E-02
**PrenatalMarijuanaCat_Num**	SportsActivitiesCat_Num	1.02	0.82	1.28	8.41E-01
**PrenatalMarijuanaCat_Num**	SleepCat_Short_Num	1.12	0.88	1.44	3.55E-01
**PrenatalMarijuanaCat_Num**	SleepDisturbanceCat_Num	1.62	1.33	1.99	2.66E-06
**PrenatalMarijuanaCat_Num**	FirstWalkAgeCat_Num	1.37	0.82	2.28	2.27E-01
**PrenatalMarijuanaCat_Num**	RollOverAgeCat_Num	0.83	0.29	2.33	7.20E-01
**PrenatalMarijuanaCat_Num**	SittingAgeCat_Num	0.63	0.34	1.18	1.50E-01
**PrenatalMarijuanaCat_Num**	FirstWordAgeCat_Num	1.00	0.70	1.43	9.86E-01
**PrenatalMarijuanaCat_Num**	PrematurityCat_Num	0.98	0.69	1.38	8.99E-01
**PrenatalMarijuanaCat_Num**	BirthweightCat_Low_Num	1.07	0.78	1.46	6.78E-01
**PrenatalMarijuanaCat_Num**	BirthweightCat_High_Num	0.92	0.63	1.33	6.51E-01
**PrenatalMarijuanaCat_Num**	MedProblemsDuringPregnancyCat_Num	1.23	0.95	1.58	1.15E-01
**PrenatalMarijuanaCat_Num**	MedProblemsDuringBirthCat_Num	0.97	0.64	1.48	8.93E-01
**PrenatalMarijuanaCat_Num**	PrenatalAlcoholCat_Num	7.68	6.13	9.61	7.50E-71
**PrenatalMarijuanaCat_Num**	PrenatalTobaccoCat_Num	9.80	7.80	12.30	6.69E-86
**PrenatalMarijuanaCat_Num**	PrenatalOtherSubstanceCat_Num	8.64	5.58	13.38	4.05E-22
**PrenatalMarijuanaCat_Num**	PhysActivity_60MinVigorous_Num	0.90	0.69	1.16	4.10E-01
**PrenatalMarijuanaCat_Num**	PhysActivity_Strengthening_Num	1.01	0.82	1.25	9.24E-01
**PrenatalMarijuanaCat_Num**	LifeTimeMedProblemsCat_Num	1.12	0.91	1.37	2.78E-01
**PrenatalMarijuanaCat_Num**	TBICat_Num	0.60	0.31	1.16	1.31E-01
**PrenatalOtherSubstanceCat_Num**	BMICategory_UW_Num	0.27	0.04	1.97	1.97E-01
**PrenatalOtherSubstanceCat_Num**	BMICategory_Obese_Num	1.26	0.79	2.00	3.29E-01
**PrenatalOtherSubstanceCat_Num**	SportsActivitiesCat_Num	0.86	0.55	1.35	5.19E-01
**PrenatalOtherSubstanceCat_Num**	SleepCat_Short_Num	1.82	1.15	2.87	1.04E-02
**PrenatalOtherSubstanceCat_Num**	SleepDisturbanceCat_Num	1.63	1.11	2.40	1.30E-02
**PrenatalOtherSubstanceCat_Num**	FirstWalkAgeCat_Num	1.40	0.56	3.52	4.68E-01
**PrenatalOtherSubstanceCat_Num**	RollOverAgeCat_Num	1.52	0.36	6.38	5.66E-01
**PrenatalOtherSubstanceCat_Num**	SittingAgeCat_Num	1.73	0.79	3.80	1.72E-01
**PrenatalOtherSubstanceCat_Num**	FirstWordAgeCat_Num	0.91	0.46	1.83	7.97E-01
**PrenatalOtherSubstanceCat_Num**	PrematurityCat_Num	1.00	0.53	1.89	9.93E-01
**PrenatalOtherSubstanceCat_Num**	BirthweightCat_Low_Num	1.02	0.56	1.86	9.50E-01
**PrenatalOtherSubstanceCat_Num**	BirthweightCat_High_Num	0.58	0.25	1.34	2.06E-01
**PrenatalOtherSubstanceCat_Num**	MedProblemsDuringPregnancyCat_Num	1.37	0.86	2.18	1.88E-01

**PrenatalOtherSubstanceCat_Num**	MedProblemsDuringBirthCat_Num	0.66	0.27	1.65	3.76E-01
**PrenatalOtherSubstanceCat_Num**	PrenatalAlcoholCat_Num	5.14	3.43	7.69	1.94E-15
**PrenatalOtherSubstanceCat_Num**	PrenatalTobaccoCat_Num	7.59	4.95	11.64	1.48E-20
**PrenatalOtherSubstanceCat_Num**	PrenatalMarijuanaCat_Num	8.70	5.64	13.43	1.46E-22
**PrenatalOtherSubstanceCat_Num**	PhysActivity_60MinVigorous_Num	0.84	0.52	1.36	4.76E-01
**PrenatalOtherSubstanceCat_Num**	PhysActivity_Strengthening_Num	0.83	0.56	1.24	3.65E-01
**PrenatalOtherSubstanceCat_Num**	LifeTimeMedProblemsCat_Num	2.07	1.41	3.05	2.13E-04
**PrenatalOtherSubstanceCat_Num**	TBICat_Num	1.59	0.68	3.68	2.83E-01
**PhysActivity_60MinVigorous_Num**	BMICategory_UW_Num	1.15	0.83	1.59	4.11E-01
**PhysActivity_60MinVigorous_Num**	BMICategory_Obese_Num	1.34	1.11	1.62	1.97E-03
**PhysActivity_60MinVigorous_Num**	SportsActivitiesCat_Num	1.27	1.08	1.50	4.62E-03
**PhysActivity_60MinVigorous_Num**	SleepCat_Short_Num	1.01	0.84	1.21	9.42E-01
**PhysActivity_60MinVigorous_Num**	SleepDisturbanceCat_Num	0.94	0.82	1.07	3.36E-01
**PhysActivity_60MinVigorous_Num**	FirstWalkAgeCat_Num	1.17	0.81	1.68	3.93E-01
**PhysActivity_60MinVigorous_Num**	RollOverAgeCat_Num	1.32	0.71	2.44	3.74E-01
**PhysActivity_60MinVigorous_Num**	SittingAgeCat_Num	1.00	0.73	1.37	9.89E-01
**PhysActivity_60MinVigorous_Num**	FirstWordAgeCat_Num	1.08	0.88	1.34	4.67E-01
**PhysActivity_60MinVigorous_Num**	PrematurityCat_Num	0.82	0.67	0.99	3.98E-02
**PhysActivity_60MinVigorous_Num**	BirthweightCat_Low_Num	0.98	0.81	1.20	8.80E-01
**PhysActivity_60MinVigorous_Num**	BirthweightCat_High_Num	1.13	0.91	1.40	2.79E-01
**PhysActivity_60MinVigorous_Num**	MedProblemsDuringPregnancyCat_Num	1.00	0.84	1.19	9.85E-01
**PhysActivity_60MinVigorous_Num**	MedProblemsDuringBirthCat_Num	0.85	0.67	1.07	1.72E-01
**PhysActivity_60MinVigorous_Num**	PrenatalAlcoholCat_Num	0.95	0.83	1.09	4.71E-01
**PhysActivity_60MinVigorous_Num**	PrenatalTobaccoCat_Num	0.90	0.75	1.09	2.93E-01
**PhysActivity_60MinVigorous_Num**	PrenatalMarijuanaCat_Num	0.88	0.68	1.14	3.22E-01
**PhysActivity_60MinVigorous_Num**	PrenatalOtherSubstanceCat_Num	0.81	0.50	1.31	3.88E-01
**PhysActivity_60MinVigorous_Num**	PhysActivity_Strengthening_Num	2.74	2.41	3.10	1.10E-55
**PhysActivity_60MinVigorous_Num**	LifeTimeMedProblemsCat_Num	1.03	0.91	1.17	6.23E-01
**PhysActivity_60MinVigorous_Num**	TBICat_Num	1.14	0.83	1.56	4.32E-01
**PhysActivity_Strengthening_Num**	BMICategory_UW_Num	1.42	1.07	1.88	1.49E-02
**PhysActivity_Strengthening_Num**	BMICategory_Obese_Num	0.99	0.86	1.13	8.30E-01
**PhysActivity_Strengthening_Num**	SportsActivitiesCat_Num	1.21	1.06	1.38	3.65E-03
**PhysActivity_Strengthening_Num**	SleepCat_Short_Num	0.97	0.84	1.13	7.25E-01
**PhysActivity_Strengthening_Num**	SleepDisturbanceCat_Num	0.98	0.88	1.09	7.26E-01
**PhysActivity_Strengthening_Num**	FirstWalkAgeCat_Num	1.14	0.85	1.52	3.74E-01
**PhysActivity_Strengthening_Num**	RollOverAgeCat_Num	0.93	0.60	1.46	7.61E-01
**PhysActivity_Strengthening_Num**	SittingAgeCat_Num	1.02	0.79	1.32	8.89E-01
**PhysActivity_Strengthening_Num**	FirstWordAgeCat_Num	1.25	1.04	1.49	1.46E-02
**PhysActivity_Strengthening_Num**	PrematurityCat_Num	1.05	0.89	1.23	5.97E-01
**PhysActivity_Strengthening_Num**	BirthweightCat_Low_Num	1.01	0.86	1.18	9.07E-01
**PhysActivity_Strengthening_Num**	BirthweightCat_High_Num	0.97	0.82	1.15	7.41E-01
**PhysActivity_Strengthening_Num**	MedProblemsDuringPregnancyCat_Num	1.19	1.03	1.37	1.72E-02
**PhysActivity_Strengthening_Num**	MedProblemsDuringBirthCat_Num	0.95	0.78	1.16	6.20E-01
**PhysActivity_Strengthening_Num**	PrenatalAlcoholCat_Num	1.07	0.95	1.20	2.74E-01
**PhysActivity_Strengthening_Num**	PrenatalTobaccoCat_Num	0.96	0.82	1.12	5.88E-01
**PhysActivity_Strengthening_Num**	PrenatalMarijuanaCat_Num	0.99	0.80	1.23	9.27E-01
**PhysActivity_Strengthening_Num**	PrenatalOtherSubstanceCat_Num	0.83	0.55	1.24	3.56E-01
**PhysActivity_Strengthening_Num**	PhysActivity_60MinVigorous_Num	2.74	2.41	3.10	1.06E-55
**PhysActivity_Strengthening_Num**	LifeTimeMedProblemsCat_Num	1.01	0.91	1.12	8.56E-01
**PhysActivity_Strengthening_Num**	TBICat_Num	1.16	0.89	1.51	2.68E-01
**LifeTimeMedProblemsCat_Num**	BMICategory_UW_Num	1.03	0.80	1.32	8.22E-01
**LifeTimeMedProblemsCat_Num**	BMICategory_Obese_Num	1.21	1.06	1.37	4.75E-03
**LifeTimeMedProblemsCat_Num**	SportsActivitiesCat_Num	0.87	0.77	0.98	2.39E-02
**LifeTimeMedProblemsCat_Num**	SleepCat_Short_Num	1.00	0.87	1.15	9.87E-01
**LifeTimeMedProblemsCat_Num**	SleepDisturbanceCat_Num	2.09	1.89	2.31	2.61E-46
**LifeTimeMedProblemsCat_Num**	FirstWalkAgeCat_Num	1.23	0.95	1.61	1.20E-01
**LifeTimeMedProblemsCat_Num**	RollOverAgeCat_Num	1.26	0.83	1.92	2.75E-01
**LifeTimeMedProblemsCat_Num**	SittingAgeCat_Num	1.53	1.21	1.94	4.46E-04
**LifeTimeMedProblemsCat_Num**	FirstWordAgeCat_Num	1.32	1.13	1.55	5.12E-04
**LifeTimeMedProblemsCat_Num**	PrematurityCat_Num	1.18	1.01	1.38	3.53E-02
**LifeTimeMedProblemsCat_Num**	BirthweightCat_Low_Num	1.08	0.93	1.26	3.00E-01
**LifeTimeMedProblemsCat_Num**	BirthweightCat_High_Num	1.03	0.88	1.21	6.88E-01
**LifeTimeMedProblemsCat_Num**	MedProblemsDuringPregnancyCat_Num	1.68	1.48	1.92	5.82E-15
**LifeTimeMedProblemsCat_Num**	MedProblemsDuringBirthCat_Num	1.40	1.16	1.69	4.32E-04
**LifeTimeMedProblemsCat_Num**	PrenatalAlcoholCat_Num	1.08	0.97	1.20	1.72E-01
**LifeTimeMedProblemsCat_Num**	PrenatalTobaccoCat_Num	1.17	1.01	1.35	3.54E-02

**LifeTimeMedProblemsCat_Num**	PrenatalMarijuanaCat_Num	1.10	0.90	1.35	3.43E-01
**LifeTimeMedProblemsCat_Num**	PrenatalOtherSubstanceCat_Num	2.05	1.39	3.01	2.70E-04
**LifeTimeMedProblemsCat_Num**	PhysActivity_60MinVigorous_Num	1.03	0.91	1.17	6.14E-01
**LifeTimeMedProblemsCat_Num**	PhysActivity_Strengthening_Num	1.01	0.91	1.12	8.53E-01
**LifeTimeMedProblemsCat_Num**	TBICat_Num	1.33	1.05	1.68	1.71E-02
**TBICat_Num**	BMICategory_UW_Num	0.51	0.22	1.15	1.05E-01
**TBICat_Num**	BMICategory_Obese_Num	0.95	0.67	1.35	7.88E-01
**TBICat_Num**	SportsActivitiesCat_Num	0.92	0.67	1.27	6.04E-01
**TBICat_Num**	SleepCat_Short_Num	1.19	0.83	1.68	3.43E-01
**TBICat_Num**	SleepDisturbanceCat_Num	1.85	1.46	2.35	4.29E-07
**TBICat_Num**	FirstWalkAgeCat_Num	1.24	0.68	2.26	4.77E-01
**TBICat_Num**	RollOverAgeCat_Num	1.22	0.44	3.37	7.06E-01
**TBICat_Num**	SittingAgeCat_Num	1.64	0.99	2.70	5.33E-02
**TBICat_Num**	FirstWordAgeCat_Num	1.12	0.77	1.64	5.54E-01
**TBICat_Num**	PrematurityCat_Num	1.10	0.75	1.60	6.37E-01
**TBICat_Num**	BirthweightCat_Low_Num	1.07	0.73	1.57	7.39E-01
**TBICat_Num**	BirthweightCat_High_Num	1.11	0.76	1.63	5.79E-01
**TBICat_Num**	MedProblemsDuringPregnancyCat_Num	1.54	1.14	2.08	4.78E-03
**TBICat_Num**	MedProblemsDuringBirthCat_Num	1.85	1.25	2.72	1.87E-03
**TBICat_Num**	PrenatalAlcoholCat_Num	0.99	0.76	1.29	9.57E-01
**TBICat_Num**	PrenatalTobaccoCat_Num	1.37	0.97	1.95	7.64E-02
**TBICat_Num**	PrenatalMarijuanaCat_Num	0.62	0.32	1.18	1.46E-01
**TBICat_Num**	PrenatalOtherSubstanceCat_Num	1.54	0.67	3.59	3.12E-01
**TBICat_Num**	PhysActivity_60MinVigorous_Num	1.13	0.82	1.56	4.42E-01
**TBICat_Num**	PhysActivity_Strengthening_Num	1.16	0.90	1.51	2.57E-01
**TBICat_Num**	LifeTimeMedProblemsCat_Num	1.33	1.05	1.68	1.69E-02
